# Surface Optimization of Noble‐Metal‐Free Conductive [Mn_1/4_Co_1/2_Ni_1/4_]O_2_ Nanosheets for Boosting Their Efficacy as Hybridization Matrices

**DOI:** 10.1002/advs.202408948

**Published:** 2024-10-04

**Authors:** Nam Hee Kwon, Se‐Jun Kim, Tae‐Ha Gu, Jang Mee Lee, Myung Hwa Kim, Dooam Paik, Xiaoyan Jin, Hyungjun Kim, Seong‐Ju Hwang

**Affiliations:** ^1^ Department of Materials Science and Engineering College of Engineering Yonsei University Seoul 03722 Republic of Korea; ^2^ Department of Chemistry Korea Advanced Institute of Science and Technology (KAIST) Daejeon 34141 Republic of Korea; ^3^ Department of Chemistry and Nanoscience College of Natural Science Ewha Womans University Seoul 03760 Republic of Korea; ^4^ Centre for Advanced Materials and Industrial Chemistry (CAMIC) School of Science Royal Melbourne Institute of Technology (RMIT) University Melbourne VIC 3001 Australia; ^5^ Department of Applied Chemistry College of Natural Science University of Seoul Seoul 02504 Republic of Korea; ^6^ Department of Battery Engineering Yonsei University Seoul 03722 Republic of Korea

**Keywords:** hybridization matrix, interfacial coordination bonding, noble‐metal‐free conductive oxide nanosheet, photo‐/electro‐catalyst, surface optimization

## Abstract

Conductive 2D nanosheets have evoked tremendous scientific efforts because of their high efficiency as hybridization matrices for improving diverse functionalities of nanostructured materials. To address the problems posed by previously reported conductive nanosheets like poorly‐interacting graphene and cost‐ineffective RuO_2_ nanosheets, economically feasible noble‐metal‐free conductive [Mn_x_Co_1−2x_Ni_x_]O_2_ oxide nanosheets are synthesized with outstanding interfacial interaction capability. The surface‐optimized [Mn_1/4_Co_1/2_Ni_1/4_]O_2_ nanosheets outperformed RuO_2_/graphene nanosheets as hybridization matrices in exploring high‐performance visible‐light‐active (λ >420 nm) photocatalysts. The most efficient g‐C_3_N_4_–[Mn_1/4_Co_1/2_Ni_1/4_]O_2_ nanohybrid exhibited unusually high photocatalytic activity (NH_4_^+^ formation rate: 1.2 mmol g^−1^ h^−1^), i.e., one of the highest N_2_ reduction efficiencies. The outstanding hybridization effect of the defective [Mn_1/4_Co_1/2_Ni_1/4_]O_2_ nanosheets is attributed to the optimization of surface bonding character and electronic structure, allowing for improved interfacial coordination bonding with g‐C_3_N_4_ at the defect sites. Results from spectroscopic measurements and theoretical calculations reveal that hybridization helps optimize the bandgap energy, and improves charge separation, N_2_ adsorptivity, and surface reactivity. The universality of the [Mn_1/4_Co_1/2_Ni_1/4_]O_2_ nanosheet as versatile hybridization matrices is corroborated by the improvement in the electrocatalytic activity of hybridized Co−Fe‐LDH as well as the photocatalytic hydrogen production ability of hybridized CdS.

## Graphical abstract

A rational design method for the synthesis of noble‐metal‐free conductive metal oxide nanosheets is developed for versatile hybridization matrices to develop economical and efficient photo‐/electro‐catalyst materials. The hybridization effect of the optimized [Mn_x_Co_1−2x_Ni_x_]O_2_ nanosheets is maximized due to fine control of surface bonding character and defect structure, enabling to form interfacial coordination bonding with catalyst materials.

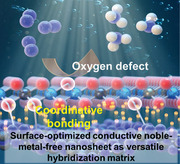

## 1 Introduction

**Figure 1:**
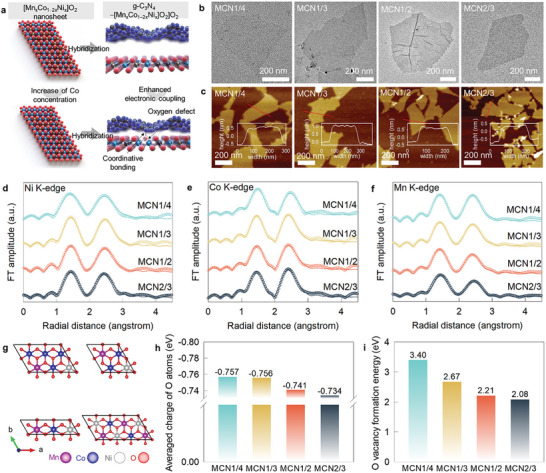
a) Schematic model for the effect of composition control on the electronic, defect, and bonding structures of [Mn_x_Co_1−2x_Ni_x_]O_2_ nanosheets. b) TEM, c) AFM, d) Ni K‐edge FT‐EXAFS, e) Co K‐edge FT‐EXAFS, f) Mn K‐edge FT‐EXAFS, g) DFT‐optimized structures, h) average charge of O atoms, and i) oxygen vacancy formation energies of [Mn_x_Co_1−2x_Ni_x_]O_2_ nanosheets.

2D conductive nanosheets are being increasingly researched as they are characterized by versatile functionalities as catalytically active materials and hybridization matrices for efficient hybrid catalysts.^[^ ^1^, ^2^, ^3^, ^4^ ^]^ However, it has been reported that the hydrophobic surface and strong self‐stacking property of graphene prevent the optimization of the hybridization effect.^[^ ^5^, ^6^ ^]^ As an emerging 2D conductive nanosheet, RuO_2_ nanosheet has been widely used owing to its high electrical conductivity and outstanding efficacy as a hybridization matrix even at low concentrations (<1 wt.%)^[^ ^7^, ^8^, ^9^ ^]^ Despite its excellent electrical conductivity and high efficacy as a hybridization matrix, the widespread use of RuO_2_ nanosheets is hindered by the scarcity and high cost of noble ruthenium.^[^ ^9^ ^]^ It is hence necessary to develop a synthetic route for the fabrication of cost‐efficient conductive oxide nanosheets. Considering the high compositional tunability of exfoliated transition metal oxide nanosheets,^[^ ^10^ ^]^ diversification of the cationic composition can help in improving the electrical conductivity, introducing anion vacancies, increasing the surface polarity, and optimizing the band structure. This results in fine‐tuning of the defect structure and surface bonding properties, which help in effectively reinforcing the interfacial chemical interaction with hybridized species via the formation of coordinative bonds and strengthening of polar dipole−dipole interactions (**Figure** **1**a).^[^ ^11^ ^]^ As hybridization with conductive nanosheets enables the suppression of charge recombination and results in increased light absorptivity and improved charge transport and kinetics, there is significant scope to explore efficient 2D hybrid photocatalysts using [Mn_x_Co_1−2x_Ni_x_]O_2_ nanosheets.^[^ ^12^ ^]^ In addition, hybridization with [Mn_x_Co_1−2x_Ni_x_]O_2_ nanosheets is also supposed to offer useful opportunity to explore high‐performance electrocatalysts, as the electronic coupling with conductive nanosheet would be quite helpful in enhancing the charge transport property and electrochemical activity. Despite the remarkable advantages offered by conductive metal oxide nanosheets,^[^ ^13^ ^]^ at the time of submission of this manuscript, there are no other papers reporting the exfoliation of surface‐tailored multicomponent transition‐metal‐based conductive [Mn_x_Co_1−2x_Ni_x_]O_2_ nanosheets and their applications as hybridization matrices.

We develop a synthetic methodology of noble‐metal‐free conductive oxide nanosheets by diversifying the cationic composition. The efficiency of [Mn_x_Co_1−2x_Ni_x_]O_2_ nanosheets as hybridization matrices is investigated by examining the impact of hybridization on the photocatalyst functionality of g‐C_3_N_4_ and CdS by conducting a visible‐light‐induced N_2_ reduction reaction (NRR) and H_2_ evolution reaction (HER). The changes in the defect structure, band structure, bond polarity, surface hydrophilicity, and electrical conductivity of [Mn_x_Co_1−2x_Ni_x_]O_2_ nanosheets with changes in the composition are systematically investigated using the theoretical and spectroscopic analyses and their effects on the catalyst performance was studied. The universal value of [Mn_x_Co_1−2x_Ni_x_]O_2_ nanosheets as versatile hybridization matrices is further examined by monitoring the impact of hybridization on the electrocatalyst performance of Co−Fe‐layered double hydroxide (LDH) nanosheets.

## 2 Results and Discussion

### 2.1 Synthesis and Chemical Features of Exfoliated [Mn_x_Co_1−2x_Ni_x_]O_2_ Nanosheets

Host layered [Mn_x_Co_1−2x_Ni_x_]O_2_ (1/4 ≤ Co content (1−2x) ≤2/3) materials were synthesized by a sol‐gel‐based solid‐state reaction. The materials were subsequently exfoliated to form monolayer nanosheets via sequential proton exchange and tetramethylammonium intercalation. The resulting 2D [Mn_x_Co_1−2x_Ni_x_]O_2_ nanosheets were labeled as **MCN1/4**, **MCN1/3**, **MCN1/2**, and **MCN2/3**, where the Co contents of 1−2x are 1/4, 1/3, 1/2, and 2/3, respectively. Based on the previous report about the synthesis of exfoliated [Mn_1/3_Co_1/3_Ni_1/3_]O_2_ nanosheets,^[^ ^12^ ^]^ we developed a composition control approach to control the surface property and defect structure of these nanosheets.

As shown in Figure S1 and Table S1 (Supporting Information), phase‐pure Li[Mn_x_Co_1−2x_Ni_x_]O_2_ materials and their protonated derivatives were synthesized, as verified by powder X‐ray diffraction (XRD). The formation of monolayer [Mn_x_Co_1−2x_Ni_x_]O_2_ nanosheets was confirmed using transmission electron microscopy (TEM) and atomic force microscopy (AFM), see Figure 1b,c. As presented in Figure S2 and Table S2 (Supporting Information), the AFM analysis for several nanosheet crystallites demonstrated that all [Mn_x_Co_1−2x_Ni_x_]O_2_ nanosheets possessed average thickness of ≈1 nm.

Results obtained using X‐ray absorption near‐edge structure (XANES) analysis (Figure S3, Supporting Information) demonstrated the stabilization of the Ni^2+^, Co^3+^, and Mn^4+^ oxidation states in [Mn_x_Co_1−2x_Ni_x_]O_2_ nanosheets, as presented in Table S3 (Supporting Information), presenting the variation of Ni K‐, Co K‐, and Mn K‐edge energies upon the change of cation composition. An increase in the Co content led to a shift in the Ni K‐, Co K‐, and Mn K‐edge energies toward the low‐energy region, reflecting a reduction in the metal oxidation states. Analysis of the results obtained using extended X‐ray absorption fine structure (EXAFS) analysis (Figure 1d−f) revealed a decrease in the coordination numbers for metal−oxygen shells with an increase in the Co content. This further confirmed the generation of more oxygen vacancies (Tables S4–S6, Supporting Information). This result can be ascribed to the lower stability of Co^3+^ ions, which results in the loss of oxygen anions.^[^ ^14^ ^]^ A gradual enrichment in the number of oxygen defects upon increasing Co content was further supported by electron paramagnetic resonance (EPR) spectroscopy (Figure S4, Supporting Information).

The effects of the chemical composition on the electronic structure and oxygen vacancy formation were investigated using the density functional theory (DFT) with the DFT‐optimized structures in Figure 1g. As presented in Figure 1h, an increase in the Co concentration led to a decrease in the average charge of the oxygen atoms. This can be ascribed to the fact that the electronegativity of Co (1.9) was higher than the average electronegativities of Mn (1.5) and Ni (1.9).^[^ ^15^ ^]^ This, in turn, modifies the formation energy associated with the oxygen vacancy depending on the Co concentration. The lowest oxygen vacancy formation energy and the maximum population of the oxygen vacancies were recorded for **MCN2/3** (Figure 1i).

**Figure 2:**
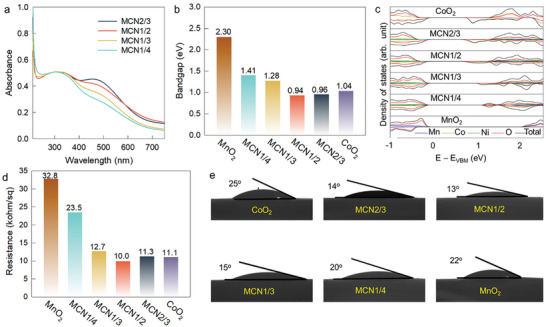
a) Diffuse reflectance UV–vis spectra, b) band gaps, c) partial density of states (PDOS) of [Mn_x_Co_1−2x_Ni_x_]O_2_ nanosheets, d) electrical conductivities, and e) contact angle images of water droplet on [Mn_x_Co_1−2x_Ni_x_]O_2_ films with several reference films.

As displayed in **Figure** **2**a, an increase in the Co content caused an increase in the peak at ≈480 nm which is assigned to the d−d transition observed in octahedral Co ions.^[^ ^12^ ^]^ Also, **MCN1/2** exhibited stronger absorption (compared to the other homologs) in the longer wavelength region (>600 nm). The results agreed well with the results obtained using the DFT (and revealed the minimized band gap energy of the **MCN1/2** system; Figure 2b,c). The electrical conductivity of [Mn_x_Co_1−2x_Ni_x_]O_2_ nanosheets was found to increase under cation diversification as a result of the increased band width and narrowed band gap; this was confirmed through four‐probe conductivity tests (Figure 2d). The **MCN1/2** nanosheet exhibited the optimized electrical conductivity with the lowest electrical resistance among all the trimetallic nanosheets under study. In addition, the superhydrophilic surface nature of [Mn_x_Co_1−2x_Ni_x_]O_2_ nanosheets under cation diversification was substantiated by measuring contact angles (Figure 2e; Figure S5a, Supporting Information).

### 2.2 Structure, Morphology, and Bonding Nature of g‐C_3_N_4_−[Mn_x_Co_1−2x_Ni_x_]O_2_ Nanohybrid

The intimately coupled g‐C_3_N_4_−[Mn_x_Co_1−2x_Ni_x_]O_2_ nanohybrids were synthesized by the electrostatically induced self‐assembly of anionic [Mn_x_Co_1−2x_Ni_x_]O_2_ nanosheets with cationic g‐C_3_N_4_ nanosheets (Figure S5b and S6, Supporting Information).^[^ ^16^ ^]^ To induce electrostatically driven self‐assembly process with [Mn_x_Co_1−2x_Ni_x_]O_2_ nanosheets, it was necessary to precisely regulate the protonation process for precursor g‐C_3_N_4_ nanosheets to change its intrinsic negative charge to positive charge without sedimentation. This process is pivotal in achieving homogenous nanoscale hybridization with negatively charged [Mn_x_Co_1−2x_Ni_x_]O_2_ nanosheets. The optimal g‐C_3_N_4_:metal oxide weight ratio was determined by preliminary composition‐dependent experiments to be 100:0.5 (FigureS7, Supporting Information). The obtained nanohybrids of g‐C_3_N_4_ nanosheets with **MCN3/2**, **MCN1/2**, **MCN1/3**, and **MCN1/4** were denoted as **CNMCN2/3**, **CNMCN1/2**, **CNMCN1/3**, and **CNMCN1/4**, respectively.

**Figure 3:**
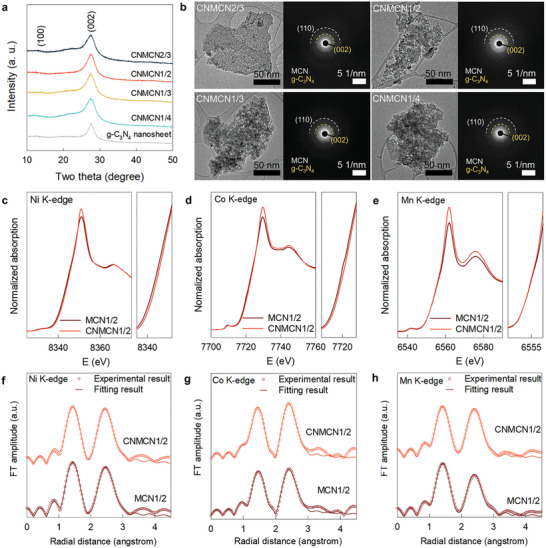
a) Powder XRD patterns and b) TEM images and SAED patterns of **CNMCN** nanohybrids with g‐C_3_N_4_ nanosheet. c) Ni K‐edge XANES, d) Co K‐edge XANES, e) Mn K‐edge XANES, f) Ni K‐edge EXAFS, g) Co K‐edge EXAFS, and h) Mn K‐edge EXAFS of **MCN1/2** nanosheet and **CNMCN1/2** nanohybrid.

All the nanohybrids exhibit typical Bragg reflections corresponding to the g‐C_3_N_4_ phase without the (*00l*) reflections of the layered metal oxide, confirming the homogeneous hybridization between two components (**Figure** **3**a). The nanoscale hybridization between [Mn_x_Co_1−2x_Ni_x_]O_2_ and g‐C_3_N_4_ nanosheets was further corroborated by the selected area electron diffraction (SAED) and elemental mapping results by energy‐dispersive spectrometry (EDS) (Figure 3b; Figure S8, Supporting Information). In Figure 3b, the observation of the in‐plane (110) peaks of hexagonal [Mn_x_Co_1−2x_Ni_x_]O_2_ lattice confirmed the maintenance of their original structure after exfoliation and hybridization processes. The (*110*) reflections observed in the SAED patterns matched well with the (*110*) peaks observed in the XRD patterns of Li[Mn_x_Co_1−2x_Ni_x_]O_2_ materials and their protonated derivatives (Figure S1, Supporting Information). This result provided additional confirmation for the retention of original hexagonal [Mn_x_Co_1−2x_Ni_x_]O_2_ lattices upon the exfoliation process.

The interfacial electron transfer in g‐C_3_N_4_−[Mn_x_Co_1−2x_Ni_x_]O_2_ nanohybrids was studied using XANES and X‐ray photoelectron spectroscopic (XPS) analyses. The Ni and Co K‐edge XANES energies of the **CNMCN1/2** nanohybrid were higher than those of the **MCN1/2** nanosheet while Mn K‐edge energy presented identical features (Figure 3c–e). This highlighted that the Co and Ni components were predominantly oxidized when the electrons were transferred into g‐C_3_N_4_ which is attributed to the lower oxidation states of Co^3+^/Ni^2+^ than Mn^4+^. Electron transfer from [Mn_x_Co_1−2x_Ni_x_]O_2_ to g‐C_3_N_4_ was cross‐confirmed by the lowering of the C 1s and N 1s binding energies upon hybridization (Figure S9, Supporting Information). As shown in Figure 3f–h, all the **CNMCN** nanohybrid revealed the original Fourier‐transformed (FT) spectral features of [Mn_x_Co_1−2x_Ni_x_]O_2_ nanosheets. Moreover, the nonlinear curve‐fitting data demonstrated that hybridization with g‐C_3_N_4_ nanosheet increased the coordination number of the metal–oxygen bonds (Tables S7–S9, Supporting Information). Since the EXAFS technique cannot be used to distinguish oxygen from nitrogen/carbon owing to their similar atomic numbers, the observed increase in the coordination numbers can be ascribed to the interfacial coordination interaction between the metal ions in [Mn_x_Co_1−2x_Ni_x_]O_2_ and carbon/nitrogen species in g‐C_3_N_4_.^[^ ^17^ ^]^

The beneficial effect of hybridization with [Mn_x_Co_1−2x_Ni_x_]O_2_ nanosheets on the porosity of the g‐C_3_N_4_ nanosheets was clearly evidenced by N_2_ adsorption–desorption isotherms measurements. As presented in Figure S10 (Supporting Information), the Brunauer–Emmett–Teller (BET) surface area and pore volume of the **CNMCN1/2** nanohybrid were larger than those of the unhybridized g‐C_3_N_4_ nanosheets, stressing the benefit of incorporating [Mn_x_Co_1−2x_Ni_x_]O_2_ nanosheets in enhancing the porosity of nanohybrid.

### 2.3 Photocatalytic Activity of the g‐C_3_N_4_−[Mn_x_Co_1−2x_Ni_x_]O_2_ Nanohybrid

**Figure 4:**
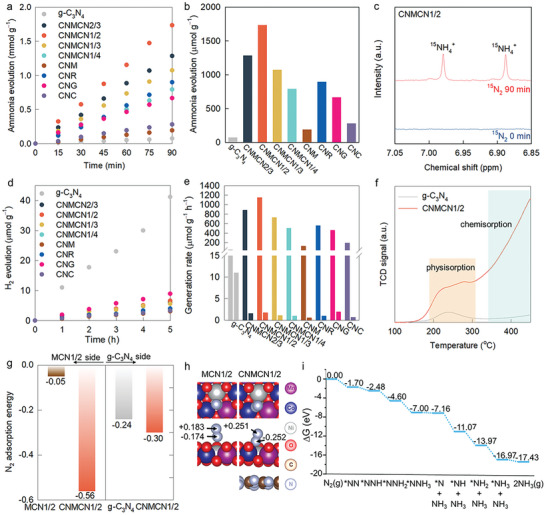
a) Visible light‐induced NRR and b) amount of evolved NH_3_ of **CNMCN** nanohybrids with several references. c) ^1^H NMR of **CNMCN1/2** nanohybrid. d) Visible light‐induced H_2_ evolution, e) generation rates of NH_3_ (left) and H_2_ (right), and f) TPD of **CNMCN1/2** nanohybrid and g‐C_3_N_4_. g) N_2_ adsorption energies on **MCN1/2**, g‐C_3_N_4_, **MCN1/2** in **CNMCN1/2**, and g‐C_3_N_4_ in **CNMCN1/2**. h) DFT‐optimized structure of N_2_ adsorbed on **MCN1/2** and **CNMCN1/2**, and the partial charges of the adsorbed N_2_ are shown. i) Free energy diagram of NRR process at the oxygen vacant site of **CNMCN1/2**, where the energy of photoexcited electron is corrected by (ε_VBM_ + *h*ν). The ε_VBM_ is DFT‐calculated valence band maximum energy and *h*ν is the photon energy for the wavelength of 420 nm.

The hybridization effect of [Mn_x_Co_1−2x_Ni_x_]O_2_ nanosheets on the photocatalytic activity was tested by conducting a visible‐light (λ >420 nm)‐induced NRR.^[^ ^18^, ^19^ ^]^ All the **CNMCN** nanohybrids exhibited superior photocatalytic NRR performances to those of g‐C_3_N_4_ and the physical mixture of **MCN1/2** nanosheet and g‐C_3_N_4_, highlighting the advantages of nanoscale hybridization (**Figure** **4**a; Figure S11, Supporting Information). Among the present nanohybrids, the maximum rate of NH_4_^+^ generation (1153 µmol g^−1^ h^−1^) was recorded for **CNMCN1/2**, which was ≈23 times greater than that of g‐C_3_N_4_ (50.4 µmol g^−1^h^−1^). This **CNMCN1/2** nanohybrid showed the best photocatalyst performance for visible‐light‐induced NRR without the use of hole scavenger among the recently reported data (Table S10, Supporting Information). Although there have been several papers reporting higher photocatalytic activity than the present material, these studies employed UV–vis radiation rather than visible light radiation. Considering the very low proportion of UV light in the solar radiation (≈4%), it is quite important to develop visible‐light‐active photocatalysts rather than UV‐active one. Furthermore, most of these studies used sacrificial agents, i.e., hole scavengers, which is not favorable for the practical application of photocatalysts. Thus, the present g‐C_3_N_4_−[Mn_x_Co_1−2x_Ni_x_]O_2_ nanohybrid could be estimated to be highly promising as practical photocatalysts for NRR. In addition, g‐C_3_N_4_ nanosheets were also hybridized with CoO_2_, MnO_2_, RuO_2_, and reduced graphene oxide (rGO) nanosheets to confirm the effective role of [Mn_x_Co_1−2x_Ni_x_]O_2_ nanosheet as depicted in Figure S12 (Supporting Information) (named as **CNC**, **CNM**, **CNR**, and **CNG**, respectively). The NRR photocatalytic activity of **CNMCN1/2** was notably higher compared to that of **CNR**, providing a compelling evidence that the **MCN1/2** nanosheet could be efficiently used as a hybridization matrix (Figure 4b).

To further corroborate the exceptional high efficiency of trimetallic [Mn_x_Co_1−2x_Ni_x_]O_2_ nanosheets as hybridization matrices, bimetallic [Mn_1/2_Co_1/2_]O_2_, [Mn_1/2_Ni_1/2_]O_2_, and [Co_1/2_Ni_1/2_]O_2_ nanosheets were also synthesized by employing the identical synthetic method to that of trimetallic homologs. Then, the obtained bimetallic nanosheets were hybridized with g‐C_3_N_4_ nanosheets, yielding the nanohybrids of g‐C_3_N_4_−[Mn_1/2_Co_1/2_]O_2_, g‐C_3_N_4_−[Mn_1/2_Ni_1/2_]O_2_, and g‐C_3_N_4_−[Co_1/2_Ni_1/2_]O_2_, see Figure S13a (Supporting Information). These materials are denoted as **CNMN**, **CNMC**, and **CNCN** respectively. Even though the hybridization with these bimetallic nanosheets led to the improvement of NRR performances than the pristine g‐C_3_N_4_, their NRR activity was found to be inferior to that of trimetallic **CNMCN1/2**, as plotted in Figure S13b (Supporting Information). These results highlighted that the precise control of cationic composition in trimetallic oxide nanosheets allowed to maximize the impact of hybridization to boost the photocatalytic activity of hybridized g‐C_3_N_4_.

The reduction of atmospheric N_2_ caused by **CNMCN1/2** was verified by detecting the formation of ^15^NH_4_^+^ in a ^15^N_2_ isotope atmosphere, while the absence of NH_4_^+^ production in a N_2_‐free argon atmosphere provided additional evidence for the NRR (Figure 4c; Figure S14, Supporting Information).^[^ ^16^, ^18^ ^]^ All the materials were also tested for the visible‐light‐induced HER (a reaction that competes with the NRR) to examine the selectivity (Figure 4d). The selectivity for the NRR exhibited by **CNMCN1/2** was higher than those recorded for **CNR** and **CNG** (Figure 4e), underscoring that hybridization with [Mn_x_Co_1−2x_Ni_x_]O_2_ nanosheets increased the selectivity.

The 2D conductive [Mn_x_Co_1−2x_Ni_x_]O_2_ nanosheets were supposed to play versatile roles in the hybrid‐type photocatalysts, such as (i) adsorbent, (ii) charge reservoir, (iii) photosensitizer, (iv) cocatalyst, and (v) charge transport pathway.^[^ ^6^ ^]^ These functions of [Mn_x_Co_1−2x_Ni_x_]O_2_ nanosheets were systematically examined to elucidate the underlying mechanism for the hybridization impact of these materials on the photocatalyst performance.^[^ ^20^ ^]^ First, the beneficial role of [Mn_x_Co_1−2x_Ni_x_]O_2_ nanosheets as (i) adsorbent was examined using temperature‐programmed desorption (TPD) and DFT calculation. As depicted in TPD plots, the results demonstrated that improved physical and chemical adsorption of N_2_ could be achieved for **CNMCN1/2** nanohybrid (Figure 4f). Notably, a higher desorption temperature for chemically adsorbed N_2_ molecules (350−450 °C) was recorded for the **CNMCN1/2** nanohybrid comparing to g‐C_3_N_4_. Considering the similarities between the sizes and shapes of oxygen vacancies and nitrogen atoms,^[^ ^21^ ^]^ the enhanced N_2_ adsorption realized using the oxygen‐defective [Mn_x_Co_1−2x_Ni_x_]O_2_ nanosheets demonstrated the pivotal role of oxygen vacancies in enhancing N_2_ adsorption.^[16]^ The influence of oxygen vacancy formation on the NRR activity was further supported by the DFT calculations. As the oxygen defect site was considered to be a catalytically active center (Figure 4g), the N_2_ adsorption realized on **CNMCN1/2** (−0.56 eV) was stronger than that recorded on **MCN1/2** (−0.05 eV). This could be attributed to the highly polar interactions between N_2_ and the metal center. This was further evidenced by the fact that the partial charges corresponding to N_2_ on **CNMCN1/2** were more polarized than those corresponding to N_2_ on **MCN1/2** (Figure 4h and 4i). Although the extent of N_2_ adsorption on **CNMCN1/2** at the g‐C_3_N_4_ side was lower than that at the **MCN1/2** side (Figure 4g), the extent of N_2_ adsorption on the g‐C_3_N_4_ side of **CNMCN1/2** (−0.30 eV) was larger with respect to that on the precursor g‐C_3_N_4_ (−0.24 eV). This confirmed the beneficial effect of hybridizing with the **MCN1/2** nanosheet as N_2_ adsorbent. Second, the roles of [Mn_x_Co_1−2x_Ni_x_]O_2_ nanosheets as ii) charge reservoirs were studied using photoluminescence (PL) spectroscopy. A strong peak of g‐C_3_N_4_ nanosheet at ≈450−650 nm significantly decreased for the **CNMCN** nanohybrids, highlighting the extension of the electron–hole lifetime (Figure S15a, Supporting Information). Furthermore, the PL intensity corresponding to **CNMCN1/2** was significantly lower than those recorded for **CNR**, **CNC**, **CNM**, and **CNG**, revealing the best function of the trimetallic **MCN1/2** nanosheet as a charge reservoir. The effective interfacial charge transfer between [Mn_x_Co_1−2x_Ni_x_]O_2_ and g‐C_3_N_4_ was confirmed using time‐resolved photoluminescence (TRPL) spectroscopy (Figures S15b and S16, Supporting Information). The PL decay data were fitted using a biexponential function based on the two decay pathways. While the fast component (τ_1_) originated from the band edge emission attributable to the recombination of delocalized carriers in the internal states of g‐C_3_N_4_, the slow component (τ_2_) was ascribed to the localized carrier recombination on the surface of g‐C_3_N_4_.^[^ ^22^ ^]^ As listed in Table S11 (Supporting Information), the lifetimes of both PL components related to the band edge emission and trap state emission properties were shortened in the order of **CNMCN1/4** < **CNMCN1/3** < **CNMCN2/3** < **CNMCN1/2**. This trend for the relative efficiencies of [Mn_x_Co_1−2x_Ni_x_]O_2_ nanosheets as electron reservoirs matched well with the trend recorded for the decrease in the PL intensity. Third, the role of [Mn_x_Co_1−2x_Ni_x_]O_2_ nanosheets as (iii) photosensitizer was studied with diffuse reflectance UV–vis spectroscopy. Hybridization with [Mn_x_Co_1−2x_Ni_x_]O_2_ nanosheets resulted in a significant increase in the visible‐light absorption, indicating the excellent photosensitizing ability (Figure S17a, Supporting Information). The best photosensitizer role of the **MCN1/2** nanosheet was evidenced by the strongest visible‐light absorption of **CNMCN1/2**. The hybridization of g‐C_3_N_4_ with [Mn_x_Co_1−2x_Ni_x_]O_2_ nanosheets also induced a modified band gap structure, as confirmed by DFT calculations (Figure S17b, Supporting Information). The band edge character of **CNMCN1/2** was primarily attributed to the N unit in g‐C_3_N_4_ and Co unit in **MCN1/2** in the valence band. The edge of conduction band was primarily determined using **MCN1/2**. This brings the decrease in the band gap of **CNMCN1/2** by 0.41 eV with respect to the unhybridized **MCN1/2**. The high efficiency of [Mn_x_Co_1−2x_Ni_x_]O_2_ nanosheet as a photosensitizer was further confirmed by hybridizing the material with the semiconducting TiO_2_ characterized by a wide band gap. As shown in Figure S17c (Supporting Information), the TiO_2_–[Mn_x_Co_1−2x_Ni_x_]O_2_ nanohybrid exhibited notable visible‐light photocatalytic HER activity, whereas bare TiO_2_ was non‐responsible toward visible‐light, indicating the effective photosensitizing ability of [Mn_x_Co_1−2x_Ni_x_]O_2_ nanosheet.

Fourth, the efficiency of [Mn_x_Co_1−2x_Ni_x_]O_2_ nanosheets as cocatalyst iv) was investigated by depositing these nanosheets on the surface of the g‐C_3_N_4_ film (Figure S18a, Supporting Information). Under visible‐light irradiation on the back of these films, the [Mn_x_Co_1−2x_Ni_x_]O_2_‐nanosheet‐covered films exhibited higher NRR activities than the uncovered g‐C_3_N_4_ film (Figure S18b, Supporting Information). This indicated the efficient role of [Mn_x_Co_1−2x_Ni_x_]O_2_ nanosheets as cocatalysts. Fifth, the efficiency of photogenerated hole and electron migrating pathway v) was probed with electrochemical impedance spectroscopy (EIS). The **CNMCN** nanohybrids exhibited significantly better charge transport properties than the **CNC** and **CNM** systems, as characterized in Figure S18c (Supporting Information). Moreover, **CNMCN1/2** and **CNMCN2/3** exhibited smaller semicircles compared to **CNR**, highlighting that the [Mn_x_Co_1−2x_Ni_x_]O_2_ nanosheet was better as a charge transport pathway than the RuO_2_ nanosheet.

The systematic investigation discussed above clearly demonstrated that the 2D conductive [Mn_x_Co_1−2x_Ni_x_]O_2_ nanosheets could deliver superior functions of adsorbent, charge reservoir, photosensitizer, cocatalyst, and charge transport pathway over the other metal oxide nanosheets including RuO_2_. Such outstanding roles of [Mn_x_Co_1−2x_Ni_x_]O_2_ nanosheets in enhancing the photocatalyst performance of hybrid material are strongly dependent on the electronic coupling with g‐C_3_N_4_ and CdS photocatalysts. The introduction of oxygen vacancies in the [Mn_x_Co_1−2x_Ni_x_]O_2_ materials helped reinforce the electronic interaction by the formation of interfacial coordination bonding with hybridized photocatalysts at the coordinatively unsaturated defect sites. The formation of interfacial bonding could enhance the interfacial charge transfer in terms of inner sphere mechanism, which is much more efficient than outer sphere mechanism without interfacial bonding. In addition, the enhanced surface polarity and oxygen vacancies of [Mn_x_Co_1−2x_Ni_x_]O_2_ nanosheets made additional contribution to the improvement of the photocatalytic activity via the provision of abundant surface reaction sites and the reinforcement of interfacial electronic coupling.

To obtain a deeper understanding about the impact of hybridization, the photocatalytic NRR activities of **CNMCN** nanohybrids were plotted as a function of several features of [Mn_x_Co_1−2x_Ni_x_]O_2_ nanosheets, such as cation composition, bandgap energy, coordination number, contact angle, and resistance. As illustrated in Figure S19 (Supporting Information), the photocatalytic performance was not well correlated directly to the cation composition. Instead, the photocatalytic activities of **CNMCN** nanohybrids showed good correlations to the surface polarity, oxygen defect, conductivity, and band gap energy. The control of surface polarity, oxygen content, electrical conductivity, and bandgap energy could be ascribed to the regulation of surface property and electronic structure via the diversification of cation composition.

### 2.4 Universal Validity of [Mn_x_Co_1−2x_Ni_x_]O_2_ Nanosheets as Hybridization Matrices

**Figure 5:**
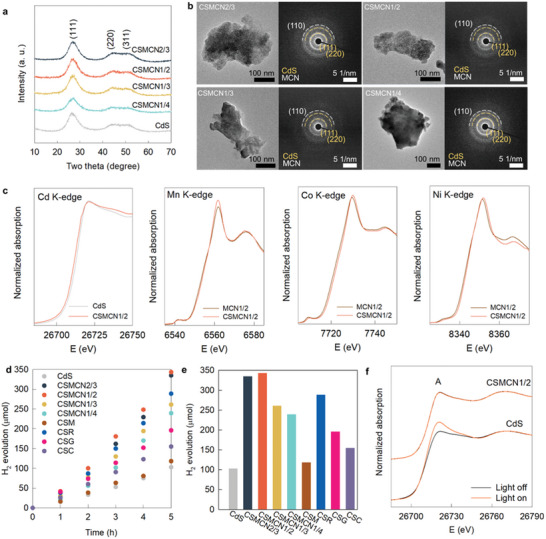
a) Powder XRD, b) TEM images and SAED patterns, c) Cd K‐edge XANES, Mn K‐edge XANES, Co K‐edge XANES, and Ni K‐edge XANES spectra of **CSMCN1/2** nanohybrid, d) visible light‐induced HER, and e) amount of evolved H_2_ of **CSMCN** nanohybrids with several references. f) Cd K‐edge XANES under visible light‐irradiation for **CSMCN1/2** nanohybrid and CdS.

To further verify the universality of [Mn_x_Co_1−2x_Ni_x_]O_2_ nanosheets as hybridization matrices, these nanosheets were also hybridized with CdS nanocrystals (Figure S5, Supporting Information), because CdS is a representative photocatalyst for HER. In addition, the CdS and g‐C_3_N_4_ showed significant differences in the surface bonding nature and polarity, which had strong influence on the interfacial interaction with [Mn_x_Co_1−2x_Ni_x_]O_2_ nanosheets. Thus, considering such dissimilar surface properties and photocatalytic applications of the CdS and g‐C_3_N_4_ materials, the examination about the hybridization impact on their photocatalytic activity could provide convincing evidence for the universal usefulness of [Mn_x_Co_1−2x_Ni_x_]O_2_ nanosheets as efficient hybridization matrices. The obtained nanohybrids composed of CdS nanocrystals with the **MCN3/2**, **MCN1/2**, **MCN1/3**, and **MCN1/4** nanosheets were designated as **CSMCN2/3**, **CSMCN1/2**, **CSMCN1/3**, and **CSMCN1/4**, respectively. The intimate hybridization between the CdS nanocrystals and [Mn_x_Co_1−2x_Ni_x_]O_2_ nanosheets was confirmed using powder XRD, HR‐TEM, and EDS‐elemental maps (**Figure** **5**a,b; Figure S20, Supporting Information). The XANES technique was used to analyze the Cd, Mn, Co, and Ni K‐edges (Figure 5c), and the results revealed that hybridization with the **MCN1/2** nanosheet resulted in a shift in the Cd K‐edge position toward the lower energy side. The energies of the Mn, Co, and Ni K‐edges recorded for **CSMCN1/2** were higher than those recorded for the **MCN1/2** nanosheet, confirming the interfacial electron transfer occurred from [Mn_x_Co_1−2x_Ni_x_]O_2_ to CdS.

As shown in Figure 5d, all the **CSMCN** nanohybrids displayed better photocatalytic visible‐light‐induced HER activities than CdS, highlighting the beneficial effect of the trimetallic [Mn_x_Co_1−2x_Ni_x_]O_2_ nanosheets. The rate of the photocatalytic HER recorded for the **CSMCN1/2** nanohybrid was estimated to be 1373 µmol g^−1^ h^−1^, which is ≈3.4 times higher compared with that of CdS (408 µmol g^−1^ h^−1^) (Figure 5e). Also, the CdS nanocrystals were hybridized with CoO_2_, MnO_2_, RuO_2_, and rGO nanosheets (denoted as **CSC**, **CSM**, **CSR**, and **CSG**, respectively) for the comparison (Figure S21, Supporting Information). Notably, **CSMCN1/2** exhibited higher HER activity than the **CSR** nanohybrid, confirming that the **MCN1/2** nanosheet could be efficiently used as a universal hybridization matrix for the photocatalysts.

The significant increase in the visible‐light‐induced photocurrent (Figure S22, Supporting Information) also reflected the advantages of forming hybrids with [Mn_x_Co_1−2x_Ni_x_]O_2_ nanosheet. Similar to the case of g‐C_3_N_4_‐based nanohybrids, the **MCN1/2** nanosheet exhibited the maximum potential to function as a charge reservoir, cocatalyst, and charge transport pathway (for CdS‐based nanohybrids) (Figure S23, Supporting Information). Notably, the photostability of CdS could be effectively improved by hybridizing the system with the **MCN1/2** nanosheet. The Cd K‐edge XANES result of CdS with/without visible‐light illumination (Figure 5f) revealed a significant increase in the resonance peak A related to the 1s → 5p transition under visible‐light illumination for pristine CdS. This indicated that the degree of covalency of the Cd─S bond decreased with an increase in the hole density of the Cd 5p orbital, reflecting the occurrence of significant structural frustration. In contrast, **CSMCN1/2** did not exhibit any spectral change in the intensity of peak A and edge energy under visible‐light irradiation, indicating that the photostability of CdS could be improved by hybridizing with **MCN1/2** nanosheets. The presence of multi‐cations improved the electrical conductivity of the [Mn_x_Co_1−2x_Ni_x_]O_2_ nanosheets by widening of band width and the narrowing of band gap energy. The increase in electrical conductivity in [Mn_x_Co_1−2x_Ni_x_]O_2_ further enhanced the impact of hybridization on the photocatalytic activity of nanohybrids.

In addition to the photocatalysts, the hybridization with [Mn_x_Co_1−2x_Ni_x_]O_2_ nanosheet was supposed to be useful in increasing the electrocatalytic activity of hybridized species. To prove the versatile high efficacy of hybridization with trimetallic nanosheets, several Co−Fe‐LDH−[Mn_x_Co_1−2x_Ni_x_]O_2_ (CFMCN) nanohybrids were synthesized ([Mn_x_Co_1−2x_Ni_x_]O_2_/Co−Fe‐LDH = 0.5, in molar ratio) and applied at oxygen evolution reaction (OER). The nanohybrids composed of Co−Fe‐LDH and **MCN3/2**, **MCN1/2**, **MCN1/3**, and **MCN1/4** were denoted as **CFMCN2/3**, **CFMCN1/2**, **CFMCN1/3**, and **CFMCN1/4**, respectively. In Figure S24a (Supporting Information), all **CFMCN** nanohybrids displayed a series of *(00l)* Bragg reflections with the in‐plane XRD peaks of Co−Fe‐LDH and [Mn_x_Co_1−2x_Ni_x_]O_2_, indicating the formation of heterostructure.^[^ ^17^ ^]^ As displayed in Figure S24b (Supporting Information), the hybridization between Co−Fe‐LDH and [Mn_x_Co_1−2x_Ni_x_]O_2_ nanosheets resulted in the creation of porous house‐of‐cards‐type stacking structure. The EDS−line scanning data of **CFMCN1/2** provided further confirmation for homogeneous mixing between Co−Fe‐LDH and **MCN1/2** nanosheets (Figure S24c, Supporting Information).

The beneficial impact of hybridization with [Mn_x_Co_1−2x_Ni_x_]O_2_ nanosheet on the electrocatalyst performance was corroborated by the OER activity measurement in the alkaline media. In Figure S25 (Supporting Information), all the **CFMCN** nanohybrids exhibited much smaller overpotentials and larger current densities with respect to those of Co−Fe‐LDH and [Mn_x_Co_1−2x_Ni_x_]O_2_ nanosheet. This result indicated the high efficiency of [Mn_x_Co_1−2x_Ni_x_]O_2_ nanosheet as hybridization matrix in enhancing the electrocatalyst functionality of hybridized LDH material. Among the **CFMCN** nanohybrids, **CFMCN1/2** presented the best performance as OER electrocatalyst, like the **CNMCN** nanohybrid. The high efficacy of the [Mn_x_Co_1−2x_Ni_x_]O_2_ nanosheet as a hybridization matrix in enhancing electrocatalysis kinetics was confirmed by a smaller Tafel slope of **CFMCN1/2** than Co−Fe‐LDH, as presented in Figure S26a and Table S12 (Supporting Information). Further, the CFMCN1/2 displayed larger electrochemical active surface areas (ECSAs) than Co−Fe‐LDH (Figure S26b and Table S12, Supporting Information). The merit of MCN1/2 nanosheet as hybridization matrix was further corroborated by the EIS data (Figure S26c and Table S12, Supporting Information) showing the smaller charge transport resistance for CFMCN1/2 than for Co−Fe‐LDH.

There are several contributing factors to the mechanism responsible for the benefit of hybridization with [Mn_x_Co_1−2x_Ni_x_]O_2_ nanosheets in improving the electrocatalytic activity. First, the high electronic conductivity of conductive [Mn_x_Co_1−2x_Ni_x_]O_2_ nanosheets enabled to facilitate the electron conduction in poorly conductive LDH due to their role of charge transport pathways. Second, the expanded surface area of exfoliated [Mn_x_Co_1−2x_Ni_x_]O_2_ nanosheets increased the ECSAs of electrocatalysts. Third, the high surface polarity of multicomponent [Mn_x_Co_1−2x_Ni_x_]O_2_ nanosheets could contribute to the promotion of electrocatalysis reactions by increasing surface reactivity. These functions of [Mn_x_Co_1−2x_Ni_x_]O_2_ nanosheets in the resulting nanohybrids were strongly dependent on the interfacial electronic coupling with LDH. The introduction of oxygen vacancies in these nanosheets could reinforce the interfacial electronic interaction via the formation of interfacial coordination bonding and the resulting dominant contribution of inner‐sphere mechanism.

## 3 Conclusion

Cost‐effective and noble‐metal‐free 2D [Mn_x_Co_1−2x_Ni_x_]O_2_ oxide nanosheets were synthesized following a scalable soft chemical exfoliation process to explore their use as efficient hybridization matrices. The trimetallic [Mn_x_Co_1−2x_Ni_x_]O_2_ nanosheet developed by us was more effective as a universal hybridization matrix than the previously reported matrices such as graphene and RuO_2_. The developed system could be used for developing high‐activity visible‐light‐driven photocatalysts. Notably, the best photocatalytic activity for the NRR (NH_4_^+^ generation rate: 1.2 mmol g^−1^ h^−1^), among all the g‐C_3_N_4_‐based materials, was recorded for the g‐C_3_N_4_−[Mn_x_Co_1−2x_Ni_x_]O_2_ nanohybrid. Although the electrical conductivity of the [Mn_x_Co_1−2x_Ni_x_]O_2_ nanosheet was lower than those of the RuO_2_ and rGO nanosheets,^[^ ^22^ ^]^ the photocatalytic activity of the [Mn_1/4_Co_1/2_Ni_1/4_]O_2_‐based nanohybrids was higher than those recorded for the RuO_2_‐/rGO‐based nanohybrids, which was ascribable to the improved extent of interfacial electronic interaction with surface‐optimized [Mn_1/4_Co_1/2_Ni_1/4_]O_2_ nanosheet. The origin of the reinforced electronic coupling in the g‐C_3_N_4_−[Mn_x_Co_1−2x_Ni_x_]O_2_ nanohybrid can be attributed to the additional interfacial coordination bonds present at the defect sites, high surface hydrophilicity/polarity, and the numerous oxygen defects in the [Mn_1/4_Co_1/2_Ni_1/4_]O_2_ nanosheet (Figure S27, Supporting Information). The unusually high efficiency of [Mn_x_Co_1−2x_Ni_x_]O_2_ nanosheets as hybridization matrices is because their function as excellent N_2_ adsorption sites, charge reservoirs, photosensitizers, charge transport pathways, and cocatalysts with optimized surface properties. In addition, hybridization with the [Mn_1/4_Co_1/2_Ni_1/4_]O_2_ nanosheet helped in effectively optimizing the photocatalyst functionality and photostability of the CdS systems during the HER. Furthermore, the universal validity of hybridization strategy presented here was further corroborated by the significant enhancement of the electrocatalytic OER performance of Co−Fe‐LDH upon coupling with [Mn_1/4_Co_1/2_Ni_1/4_]O_2_ nanosheets. Such improvement of electrocatalyst performance could be ascribed to the following contributing functions of [Mn_x_Co_1−2x_Ni_x_]O_2_ nanosheets such as a charge transport pathway, an ECSA enhancer, and an surface polarity enhancer. In summary, the marked improvement of the photocatalyst and electrocatalyst functionalities of g‐C_3_N_4_, CdS, and LDH could be attributed to the outstanding capability of defect‐introduced [Mn_x_Co_1−2x_Ni_x_]O_2_ nanosheets in enhancing interfacial electronic coupling. The introduction of anion vacancy was found to be quite effective in reinforcing the electronic interaction between hybridized species via the formation of interfacial coordination bonding and the resulting dominant contribution of inner sphere mechanism. This mechanism is mainly responsible for the universal merits of [Mn_x_Co_1−2x_Ni_x_]O_2_ nanosheets as hybridization matrices. We believe that the results reported herein can potentially help in attracting the attention of researchers studying the application of [Mn_x_Co_1−2x_Ni_x_]O_2_ nanosheets in the field of developing artificial photosynthesis catalysts and efficient water electrolyzers. Moreover, the application of the composition‐controlled [Mn_x_Co_1−2x_Ni_x_]O_2_ nanosheets as building blocks is expected to open a new chapter in the field of design and synthesis of high‐performance electrode materials that can be used to fabricate rechargeable metal ion‐based batteries (as [Mn_x_Co_1−2x_Ni_x_]O_2_ materials are one of the most efficient cathode materials used in the fabrication of Li‐ion batteries).^[^ ^23^, ^24^, ^25^, ^26^, ^27^ ^]^

## Conflict of Interest

The authors declare no conflict of interest.

## Supporting information



Supporting Information

## Data Availability

The data that support the findings of this study are available from the corresponding author upon reasonable request.
