# Supplementary material for: Surface Optimization of Noble‐Metal‐Free Conductive [Mn1/4Co1/2Ni1/4]O2 Nanosheets for Boosting Their Efficacy as Hybridization Matrices
Source: Adv Sci (Weinh). 2024 Oct 4;11(44):2408948. doi: 10.1002/advs.202408948 (PMC11600246; doi:10.1002/advs.202408948)
Supplement: Supplementary file 1 — Supporting Information [file ADVS-11-2408948-s001.docx]

Supporting Information for

Surface Optimization of Noble-Metal-Free Conductive [Mn_1/4_Co_1/2_Ni_1/4_]O_2_ Nanosheets for Boosting Their Efficacy as Hybridization Matrices

Nam Hee Kwon, Se-Jun Kim, Tae-Ha Gu, Jang Mee Lee, Myung Hwa Kim, Dooam Paik, Xiaoyan Jin,* Hyungjun Kim,* and Seong-Ju Hwang*

Experimental Section

*Synthesis*: The precursors Li[Mn_x_Co_1−2x_Ni_x_]O_2_ (Co content (1−2x) = 1/4, 1/3, 1/2, and 2/3) were synthesized by solid-state reaction assisted by a sol-gel reaction. The precursors were then reacted with a solution of HCl (1 M) to exchange the interlayer lithium ions with protons.^[1]^ The exfoliated nanosheets of the layered [Mn_x_Co_1−2x_Ni_x_]O_2_ materials were prepared by reacting the protonated Li[Mn_x_Co_1−2x_Ni_x_]O_2_ materials with tetramethylammonium ions (>10 days) at low temperatures. The positively charged g-C_3_N_4_ nanosheets were prepared by the thermally driven polycondensation of melamine precursor. The resulting material was reacted with a solution of H_2_SO_4_ for 0.5 h.^[2]^ Then, the synthesized powder was washed with distilled water and subjected to sonication for 5 h to achieve exfoliation. Positively charged g-C_3_N_4_ nanosheets were obtained by controlling the suspension pH to 3. A colloidal suspension of [Mn_x_Co_1−2x_Ni_x_]O_2_ was slowly added to the g-C_3_N_4_ nanosheet for hybridization. Following completion of the reaction, the precipitates of nanohybrids were recovered by centrifugation, and the recovered material was washed with distilled water and freeze-dried. For the nanohybrid system consisting of CdS−[Mn_x_Co_1−2x_Ni_x_]O_2_, positively charged CdS QDs (quantum dots) were synthesized by a solution-based method reported previously,^[3]^ where cadmium acetate dehydrate was reacted at 40 °C with 2-mercaptoethylamine hydrochloride and thioacetamide for 5 h. Following this, the mixture was reacted at 60 °C for 5 h under magnetic stirring. Powdered amine-anchored CdS QDs were recovered by repeatedly dispersing them in water and precipitating the desired material by adding isopropyl alcohol. The CdS−[Mn_x_Co_1−2x_Ni_x_]O_2_ nanohybrid was synthesized by dropping a colloidal suspension of exfoliated [Mn_x_Co_1−2x_Ni_x_]O_2_ nanosheets into that of CdS at 25 °C under stirring. Hybridization was performed at 60 °C for 3 h. The yellowish precipitate was recovered by centrifugation, washed repeatedly with distilled water, and dried in a vacuum oven at 50 °C for 1 day. For the synthesis of Co−Fe-LDH−[Mn_x_Co_1−2x_Ni_x_]O_2_ nanohybrid, positively charged Co−Fe-LDH nanosheets were prepared by a previously reported coprecipitation method followed by the liquid exfoliation method using formamide.^[4]^ The Co−Fe-LDH−[Mn_x_Co_1−2x_Ni_x_]O_2_ nanohybrid was synthesized by self-assembly methods of slow addition of colloidal suspension of [Mn_x_Co_1−2x_Ni_x_]O_2_ nanosheet to Co−Fe-LDH nanosheet ([Mn_x_Co_1−2x_Ni_x_]O_2_/Co−Fe-LDH=0.5, in molar ratio). The prepared precipitates were thoroughly washed with ethanol and deionized water, and freeze-dried.

*Characterization:* The crystal phases of the present materials were identified using powder XRD (Rigaku Ultima IV, λ = 1.5418 Å). The crystal shapes and hybrid structures of the nanohybrids were characterized with TEM (JEM-F200, Jeol). EDS−elemental mapping was performed to analyze the elemental distribution of the present materials using an energy-dispersive X-ray spectrometer installed with a field emission-scanning electron microscopy (FE-SEM) apparatus (JSM-7001F, Jeol). The nature of chemical bonds (for the nanohybrid) was investigated using the XANES/EXAFS technique. The Mn K-, Co K-, Ni K-, and Cd K-edge spectral profiles were recorded in the EXAFS facility equipped at beamline 10C at the Pohang Accelerator Laboratory (PAL, Korea). The XANES/EXAFS spectra were recorded in the transmission mode by employing gas ionization chambers as detectors. Prior to the measurements, the thin films were made on tapes with inert silicon oil. All data were recorded at 25 °C with a Si (111) single-crystal monochromator. No focusing mirror was utilized. The energy calibration for the measured XANES spectra was conducted by simultaneously collecting the spectra of Mn/Co/Ni/Cd metal foil. Ifeffit (version 1.2.9.) was used for the correction of background signal. It was also used to normalize the spectral profiles. The electrical resistance of the metal-oxide-nanosheet-based films was evaluated using the four-point probe measurement. Contact angles of present materials were measured for a water droplet using a DSA 100 (KRÜSS) instrument. The electronic structures of the materials were investigated using diffuse reflectance UV−vis and PL spectroscopies. The diffuse reflectance UV−vis spectral profiles were recorded on a JASCO V-760 spectrometer. An integrating sphere with BaSO_4_ was used as a reference to estimate the optical properties and band structures of present materials. The PL spectral profiles of the materials were recorded using a Perkin Elmer FL8500 fluorescence spectrometer and collected with the g-C_3_N_4_− [Mn_x_Co_1−2x_Ni_x_]O_2_ and CdS−[Mn_x_Co_1−2x_Ni_x_]O_2_ nanohybrids at excitation wavelengths 365 nm. The TRPL spectroscopy (MicroTime-200) analysis was conducted to estimate the lifetimes of the PL components in the nanohybrids.

*Photocatalytic reactivity tests.* The photocatalytic NRR activity was characterized using a Pyrex cell and a Newport Xe lamp with a power of 300 W. A cutoff filter was set (λ > 420 nm). The experiments were carried out in an atmosphere of flowing N_2_. Powdery photocatalyst (30 mg) was suspended in deionized water (100 mL), and then the suspension was subjected to N_2_ bubbling for 0.5 h (in the dark) before allowing the photoreaction to proceed. One milliliter of the reacted solution was collected from the reaction cell every 15 min, and the collected sample was filtered with a help of a syringe filter having a pore size of 0.2 μm. The amount of generated NH_4_^+^ was determined using Nessler’s reagent.^[2]^ Reliable activity data for the NRR (within reasonable error ranges) could be obtained using a neutral electrolyte without a sacrificial reagent. A fixed time of 10 min was maintained for data acquisition. The ^1^H NMR data were recorded with an Ascend AVANCE III HD Bruker spectrometer (800 MHz, Bruker BioSpin AG) using a triple-resonance CPTIC cryogenic probe (5 mm). The measurement of photocatalytic HER activity of CdS−[Mn_x_Co_1−2x_Ni_x_]O_2_ nanohybrids was performed by following previous report.^[5]^ The quantity of evolved H_2_ gas was evaluated using gas chromatography (GC, Shimadzu GC-2014). The impact of hybridization on the nitrogen adsorptivity of g-C_3_N_4_ was examined by conducting TPD (Belcat II) experiments. The photocatalytic activity test was conducted with the obtained multilayer films to evaluate the cocatalyst efficiency of the [Mn_x_Co_1−2x_Ni_x_]O_2_ nanosheets. The tests were conducted to determine if photocatalytic reaction sites could be efficiently generated (as the photocatalytic NRR can only occur at the top [Mn_x_Co_1−2x_Ni_x_]O_2_ nanosheet layer). The interference produced under light absorption (by the top conductive nanosheet layer) was eliminated by irradiating visible light from the backside of the film.

*Electrocatalyst tests:* The ink of catalyst was prepared via a dispersion of catalyst material (7 mg), carbon black (Vulcan-XC72R, 3 mg), and a 5wt% Nafion solution (20 μL) in a mixture of Milli-Q water and isopropanol (5 mL, 4:1, v/v), followed by ultrasonication for 1 h. For the preparation of the working electrode, the catalyst ink (10 μL) was deposited onto a glassy carbon electrode having a 3 mm diameter (ALS Co.), which was followed by drying at 50 °C. A Pt wire and a saturated calomel electrode (SCE) were utilized as the counter and reference electrodes, respectively. 1.0 M KOH solution served as electrolyte. Prior to measurement, O_2_ bubbling was done into the electrolyte for 0.5 h. The normalization of potentials was conducted with respect to the reversible hydrogen electrode (RHE). All electrochemical measurements involving LSV, cyclic voltammetry (CV), and EIS were conducted using the IVIUM analyzer and an RRDE-3A (ALS Co.) rotator. LSV curve measurement was carried out at a constant scan rate (5 mV s⁻¹) and a rotating speed (1600 rpm). The EIS curves were recorded over a frequency range of 0.1–100000 Hz.

*DFT calculations.* The spin-polarized DFT calculations were performed using the Vienna Ab-initio Software Package (VASP).^[6]^ The projector-augmented wave (PAW) method was used to treat the core electrons. For transition metals such as Mn, Co, and Ni, the valence electrons of 3p^6^3d^6^4s^1^ (Mn), 3d^8^4s^1^ (Co), and 3d^9^4s^1^ (Ni) were considered.^[7]^ An additional vacuum of ~15 Å was considered to avoid undesirable interactions between images across the periodic boundary. The Perdew-Burke-Enzerhof (PBE) functional was used to describe the exchange and correlation behavior of electrons, and Grimme’s D3 correction method was used to accurately describe the van der Waals interaction.^[8,9]^ The energy cutoff for the wavefunction was set at 400 eV. The mesh dimensions were used to sample. The reciprocal spaces of various [Mn_x_Co_1−2x_Ni_x_]O_2_ compounds and g-C_3_N_4_ are tabulated in Table S13. A (1×2×1) k-point mesh with the Monkhorst–Pack scheme was used to determine the **CNMCN1/2** structure.^[10]^ Hubbard corrections were applied with U_eff_ values of 5.10 eV for Mn, 3.00 eV for Co, and 5.96 eV for Ni.^[11,12]^ The partial charge distribution was analyzed using the Bader charge scheme.^[13]^ The oxygen vacancy formation energies $(\Delta_{f}E\left( V_{O}^{q} \right)$ of [Mn_x_Co_1−2x_Ni_x_]O_2_ and **CNMCN1/2** were calculated by removing one oxygen atom from the supercell consisting of 24 transition metal atoms and 48 oxygen atoms. $\Delta_{f}E\left( V_{O}^{q} \right)$ is defined as follows:^[14]^

$$\Delta_{f}E\left( V_{O}^{q} \right)= E\left( V_{O}^{q} \right)-E\left( \mathrm{bulk} \right)-\sum_{i} \Delta{n_{i}\mu}_{i}+q\varepsilon_{F}$$

where $E\left( V_{O}^{q} \right)$ is the DFT-calculated total energy of the defective structure, $E\left( \mathrm{bulk} \right)$ is the DFT-calculated total energy of the defect-free structure, $n_{i}$ is the change in the atom of element $i$, $\mu_{i}$ is the chemical potential of an atom of element $i$, $q$ is the charge of the defect, and $\varepsilon_{F}$ is the Fermi level. Here, a neutral defect was considered, and the chemical potential of oxygen $\mu_{o}$ was defined by the half of the DFT-calculated total energy of the O_2_ molecule.^[15]^ We used the VASPsol package and considered the dielectric constant of water (78.4) to include the solvation effect.^[15,16]^ The dimension of the mesh used for reciprocal space sampling in the density of states calculations (along the k_x_ and k_y_ directions) was twice as dense as that used for self-consistent field (SCF) calculations. Dipole correction was applied perpendicular to the slab. The free energy diagrams representing the reactions were generated by calculating the free energies by including the zero-point energy (ΔE_ZPE_) and entropic contribution (TΔS) using the harmonic oscillator partition function and experimental thermodynamic values (at 298.15 K).^[17]^ The chemical potential corresponding to the proton–electron couple was determined following the computational hydrogen electrode (CHE) method,^[16]^ in which the external bias potential was adjusted to the chemical potential of the photoexcited electron. It is represented as (ε_VBM_ + *h*ν), where ε_VBM_ is the DFT-calculated valence band maximum energy, and hν is the photon energy for the wavelength of 420 nm.


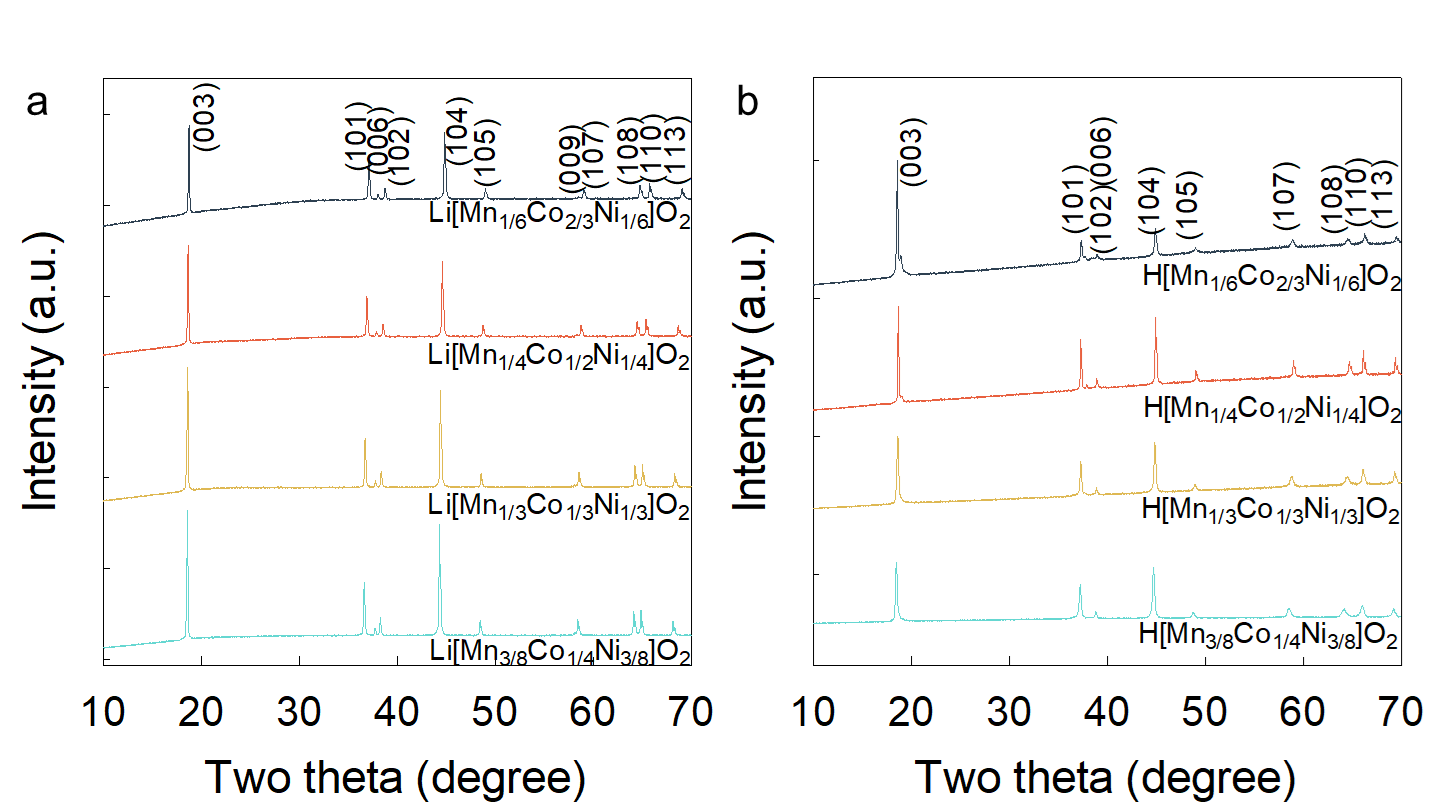


Figure S1. Powder X-ray diffraction (XRD) patterns of (a) Li[Mn_x_Co_1−2x_Ni_x_]O_2_ materials and (b) their protonated derivatives.

Table S1. Lattice parameters of Li[Mn_x_Co_1−2x_Ni_x_]O_2_ materials and their protonated derivatives.

| Material | a (Å) | c (Å) |
| --- | --- | --- |
| **H-MCN2/3** | 2.81547 | 14.29238 |
| **H-MCN1/2** | 2.82307 | 14.26030 |
| **H-MCN1/3** | 2.82311 | 14.29818 |
| **H-MCN1/4** | 2.82450 | 14.33958 |
| **Li-MCN2/3** | 2.83694 | 14.18160 |
| **Li-MCN1/2** | 2.85156 | 14.24275 |
| **Li-MCN1/3** | 2.86611 | 14.27045 |
| **Li-MCN1/4** | 2.87252 | 14.28785 |


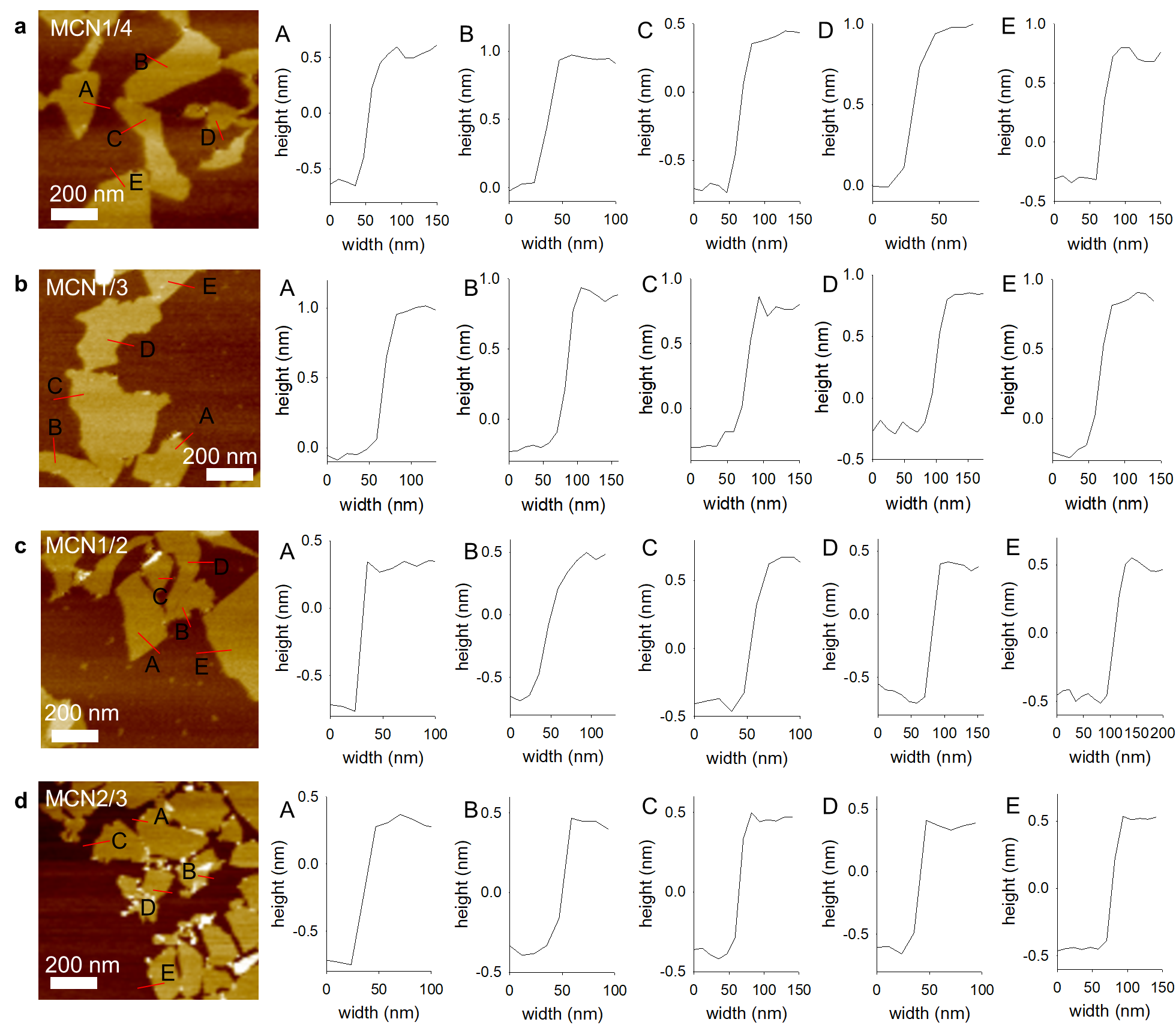


**Figure S2.** Atomic force microscopy (AFM) images and height profiles of [Mn_x_Co_1−2x_Ni_x_]O_2_ nanosheets.

**Table S2.** Layer thicknesses of [Mn_x_Co_1−2x_Ni_x_]O_2_ nanosheets measured by AFM.

| Material | A  (nm) | B  (nm) | C  (nm) | D  (nm) | E  (nm) | Average  (nm) |
| --- | --- | --- | --- | --- | --- | --- |
| **MCN2/3** | 1.01 | 0.93 | 1.00 | 0.97 | 0.97 | 0.97 |
| **MCN1/2** | 1.02 | 1.10 | 1.01 | 1.03 | 1.00 | 1.03 |
| **MCN1/3** | 1.02 | 1.04 | 1.06 | 1.04 | 1.06 | 1.04 |
| **MCN1/4** | 1.08 | 0.94 | 1.06 | 0.97 | 1.02 | 1.01 |


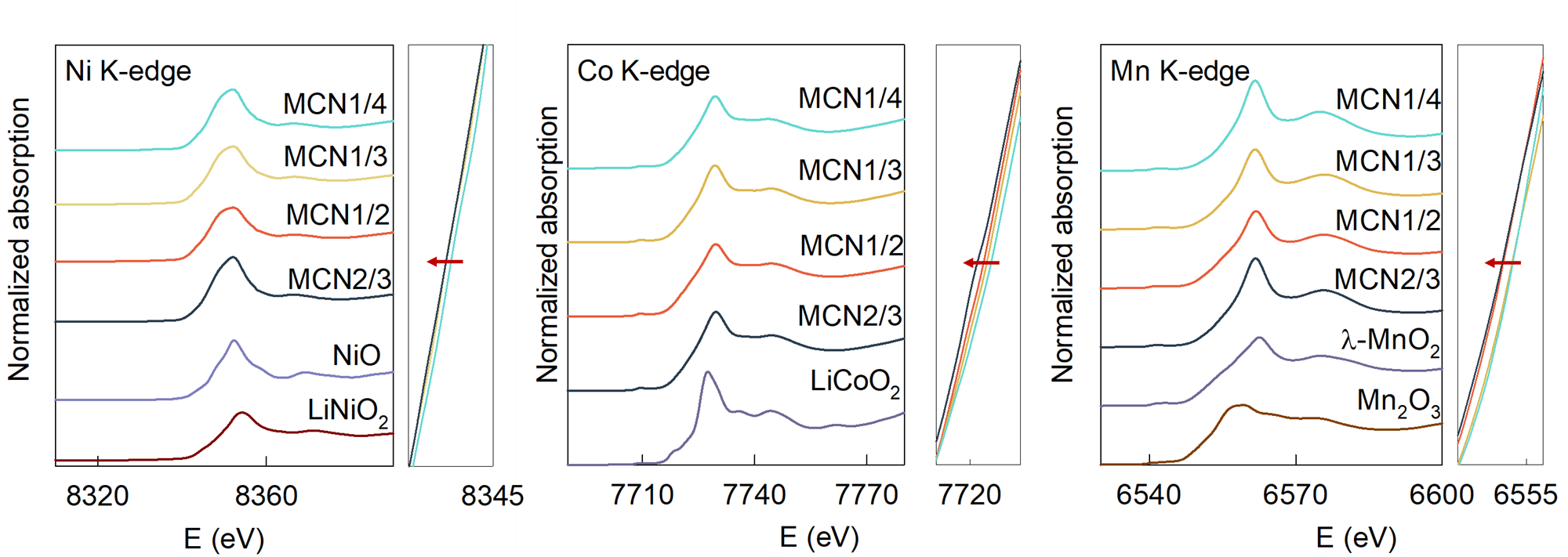


Figure S3. Ni K-edge X-ray absorption near-edge structure (XANES), Co K-edge XANES, and Mn K-edge XANES spectra of [Mn_x_Co_1−2x_Ni_x_]O_2_ nanosheets with some references.

**Table S3.** Mn K-, Co K-, and Ni K-edge energies of [Mn_x_Co_1−2x_Ni_x_]O_2_ nanosheets determined by XANES analyses.

| Material | Mn | Co | Ni |
| --- | --- | --- | --- |
| **MCN2/3** | 6552.83 | 7720.96 | 8343.35 |
| **MCN1/2** | 6553.04 | 7721.20 | 8343.42 |
| **MCN1/3** | 6553.52 | 7721.37 | 8343.59 |
| **MCN1/4** | 6553.79 | 7721.72 | 8343.87 |

Table S4. Results of non-linear least-squares curves-fittings for the Ni K-edge extended X-ray absorption fine structure (EXAFS) spectra of [Mn_x_Co_1−2x_Ni_x_]O_2_ nanosheets.

| Material | Bond | Coordination number | R (Å) | σ^2^ (10^−3^ × Å^2^) |
| --- | --- | --- | --- | --- |
| **MCN2/3** | Ni−O | 4.97 | 1.90 | 2.71 |
|  | Ni−M | 5.08 | 2.81 | 6.40 |
| **MCN1/2** | Ni−O | 5.01 | 1.90 | 3.38 |
|  | Ni−M | 5.19 | 2.82 | 7.34 |
| **MCN1/3** | Ni−O | 5.11 | 1.89 | 3.92 |
|  | Ni−M | 5.34 | 2.83 | 7.52 |
| **MCN1/4** | Ni−O | 5.49 | 1.90 | 4.29 |
|  | Ni−M | 5.95 | 2.83 | 8.85 |

Table S5. Results of non-linear least-squares curves-fittings for the Co K-edge EXAFS spectra of [Mn_x_Co_1−2x_Ni_x_]O_2_ nanosheets.

| Material | Bond | Coordination number | R (Å) | σ^2^ (10^−3^ × Å^2^) |
| --- | --- | --- | --- | --- |
| **MCN2/3** | Co−O | 4.75 | 1.90 | 2.88 |
|  | Co−M | 5.13 | 2.81 | 3.87 |
| **MCN1/2** | Co−O | 4.80 | 1.90 | 1.73 |
|  | Co−M | 5.54 | 2.81 | 4.12 |
| **MCN1/3** | Co−O | 5.24 | 1.90 | 2.38 |
|  | Co−M | 5.79 | 2.81 | 3.67 |
| **MCN1/4** | Co−O | 5.52 | 1.90 | 2.75 |
|  | Co−M | 5.95 | 2.84 | 8.45 |

Table S6. Results of non-linear least-squares curves-fittings for the Mn K-edge EXAFS spectra of [Mn_x_Co_1−2x_Ni_x_]O_2_ nanosheets.

| Material | Bond | Coordination number | R (Å) | σ^2^ (10^−3^ × Å^2^) |
| --- | --- | --- | --- | --- |
| **MCN2/3** | Mn−O | 4.92 | 1.88 | 4.07 |
|  | Mn−M | 4.99 | 2.81 | 7.91 |
| **MCN1/2** | Mn−O | 5.13 | 1.88 | 4.71 |
|  | Mn−M | 5.21 | 2.79 | 6.80 |
| **MCN1/3** | Mn−O | 5.28 | 1.88 | 3.71 |
|  | Mn−M | 5.78 | 2.79 | 7.29 |
| **MCN1/4** | Mn−O | 5.50 | 1.89 | 4.49 |
|  | Mn−M | 5.84 | 2.79 | 7.41 |


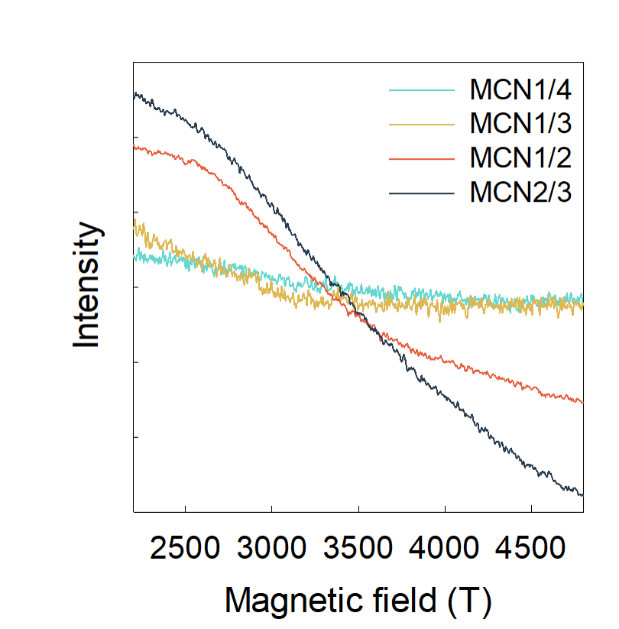


Figure S4. Electron paramagnetic resonance (EPR) spectra of [Mn_x_Co_1−2x_Ni_x_]O_2_ nanosheets.


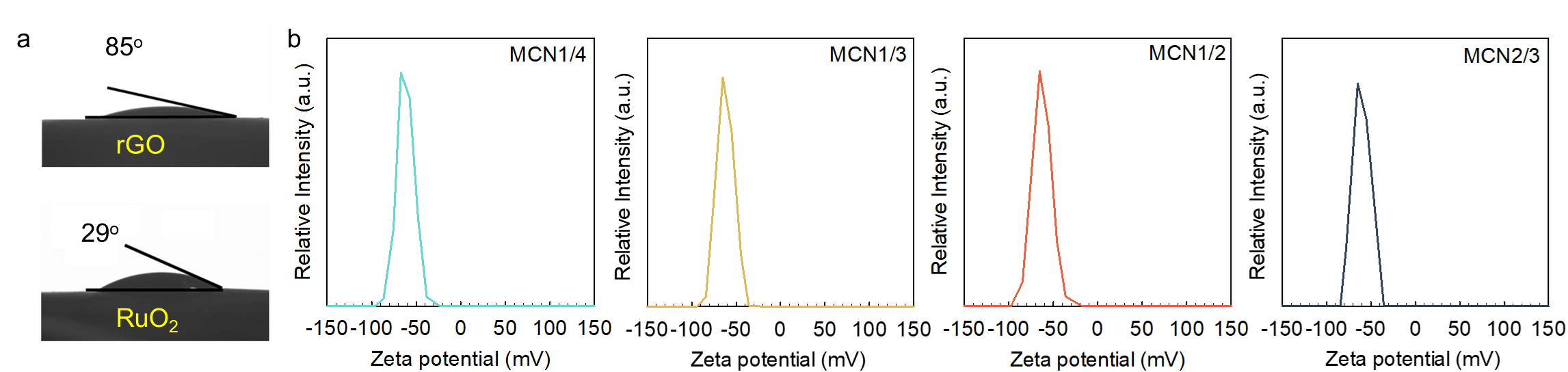


Figure S5. (a) Contact angle images of water droplet on reduced graphene oxide (rGO) and RuO_2_ films and (b) zeta potentials of [Mn_x_Co_1−2x_Ni_x_]O_2_ nanosheets.


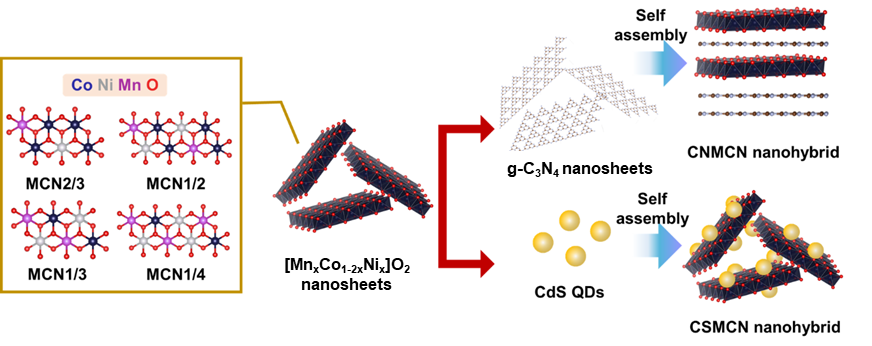


**Figure S6**. Schematic diagram of self-assembly route to [Mn_x_Co_1−2x_Ni_x_]O_2_-based nanohybrids.


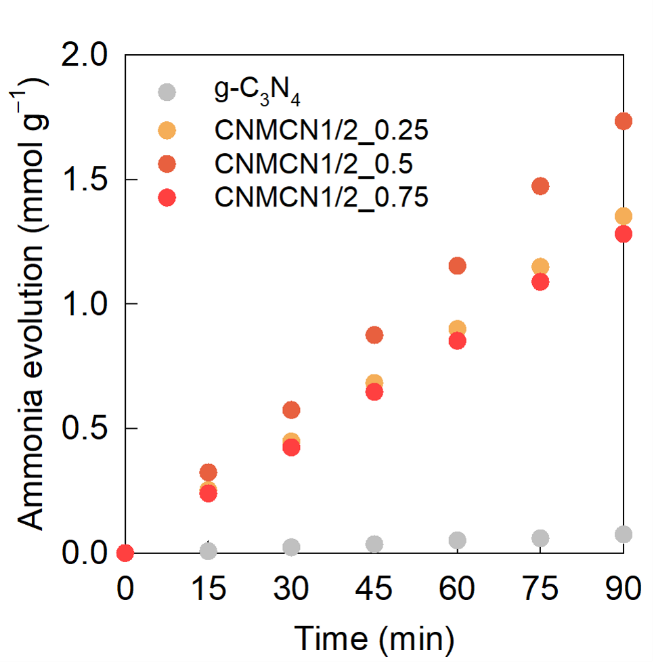


Figure S7. The preliminary composition-dependent experiments with the MCN1/2 over g-C_3_N_4_ ratios of 0.25, 0.5, and 0.75 wt%.


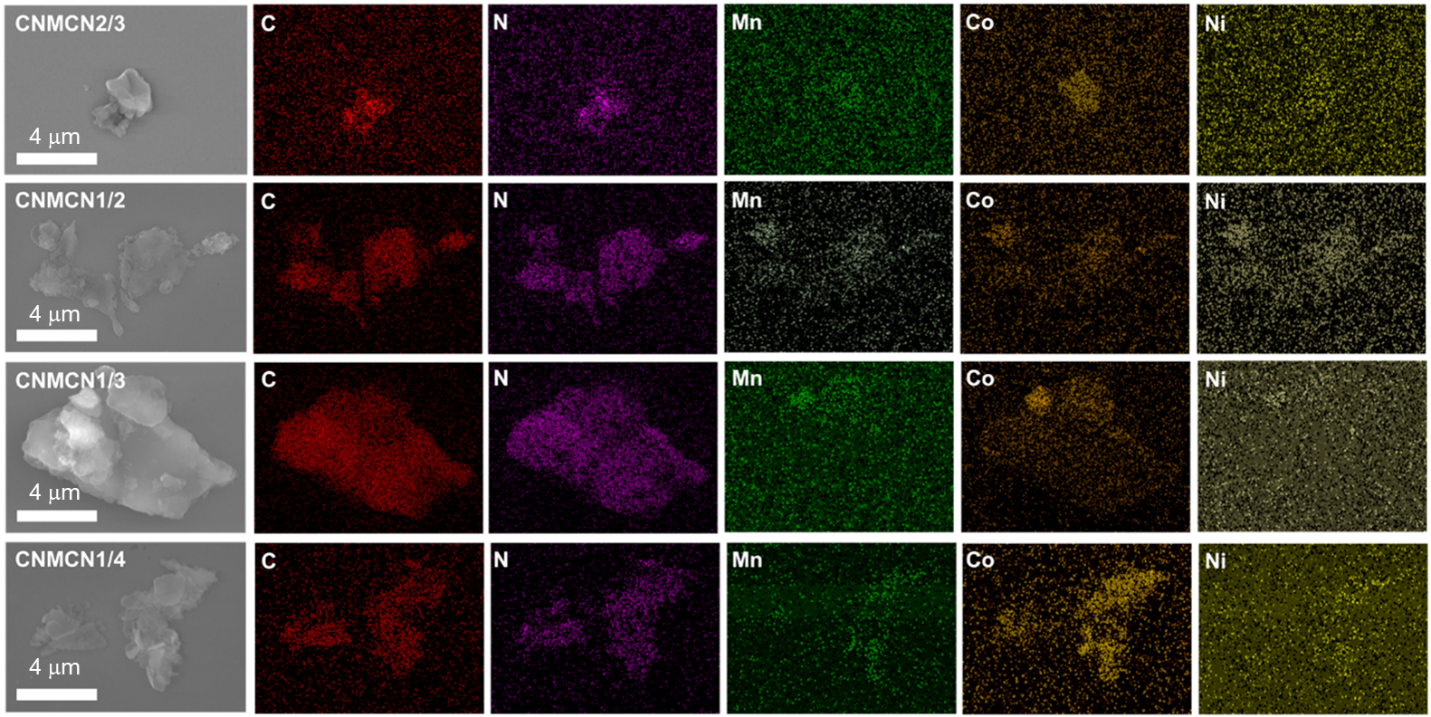


Figure S8. Energy dispersive spectrometry (EDS)–elemental maps of CNMCN nanohybrids.


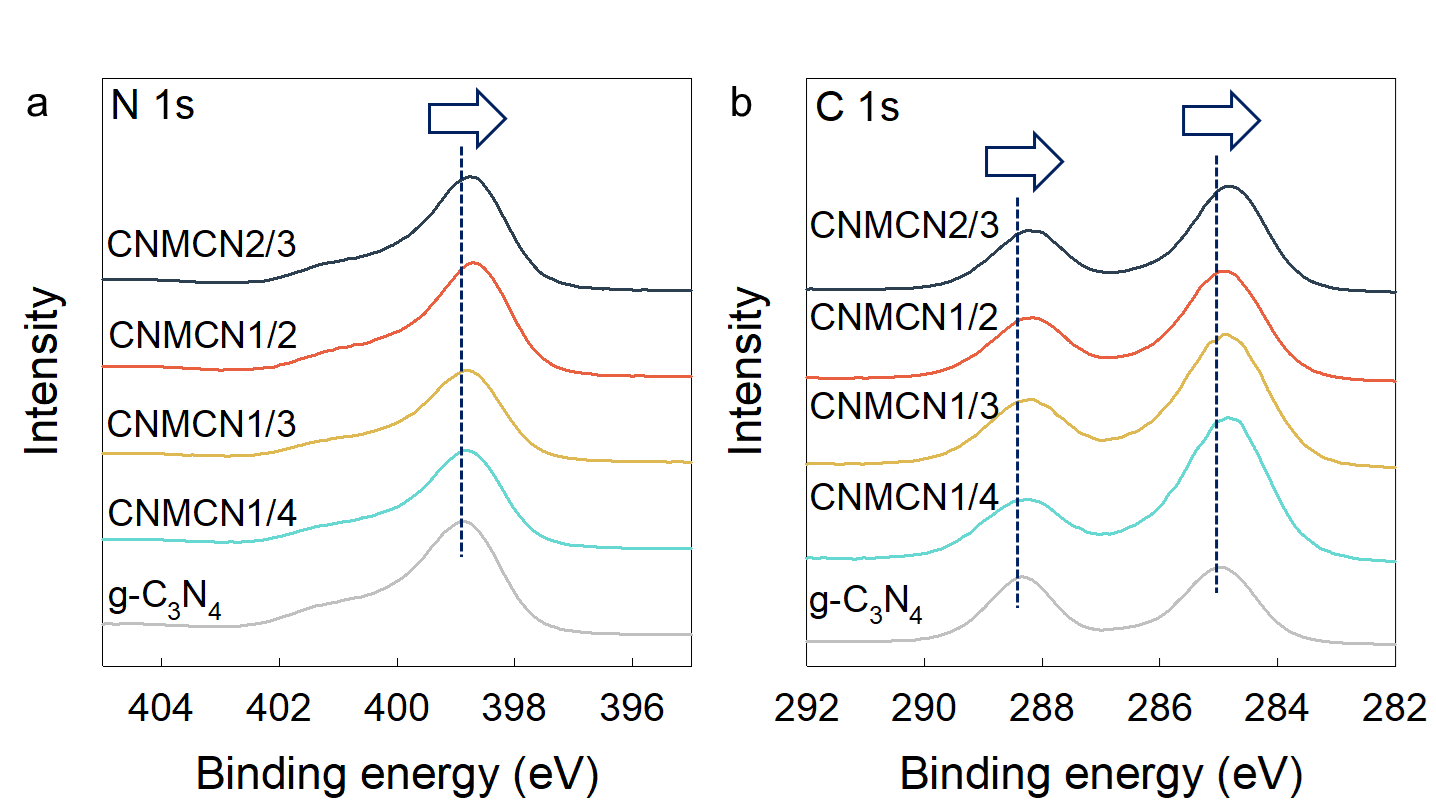


Figure S9. (a) N 1s and (b) C 1s X-ray photoelectron spectroscopy (XPS) data of CNMCN nanohybrids.

Table S7. Results of non-linear least-squares curves-fittings for the Ni K-edge EXAFS spectra of MCN1/2 nanosheet and CNMCN1/2 nanohybrid.

| Material | Bond | Coordination number | R (Å) | σ^2^ (10^−3^ × Å^2^) |
| --- | --- | --- | --- | --- |
| **CNMCN1/2** | Ni−O | 5.25 | 1.90 | 4.41 |
|  | Ni−M | 5.48 | 2.81 | 7.23 |
| **MCN1/2** | Ni−O | 5.01 | 1.90 | 3.38 |
|  | Ni−M | 5.19 | 2.82 | 7.34 |

Table S8. Results of non-linear least-squares curves-fittings for the Co K-edge EXAFS spectra of MCN1/2 nanosheet and CNMCN1/2 nanohybrid.

| Material | Bond | Coordination number | R (Å) | σ^2^ (10^−3^ × Å^2^) |
| --- | --- | --- | --- | --- |
| **CNMCN1/2** | Co−O | 5.34 | 1.89 | 3.58 |
|  | Co−M | 5.63 | 2.80 | 4.96 |
| **MCN1/2** | Co−O | 4.80 | 1.90 | 1.73 |
|  | Co−M | 5.54 | 2.81 | 4.12 |

Table S9. Results of non-linear least-squares curves-fittings for the Mn K-edge EXAFS spectra of MCN1/2 nanosheet and CNMCN1/2 nanohybrid.

| Material | Bond | Coordination number | R (Å) | σ^2^ (10^−3^ × Å^2^) |
| --- | --- | --- | --- | --- |
| **CNMCN1/2** | Mn−O | 5.21 | 1.88 | 5.74 |
|  | Mn−M | 5.65 | 2.79 | 7.82 |
| **MCN1/2** | Mn−O | 5.13 | 1.88 | 4.71 |
|  | Mn−M | 5.21 | 2.79 | 6.80 |

**
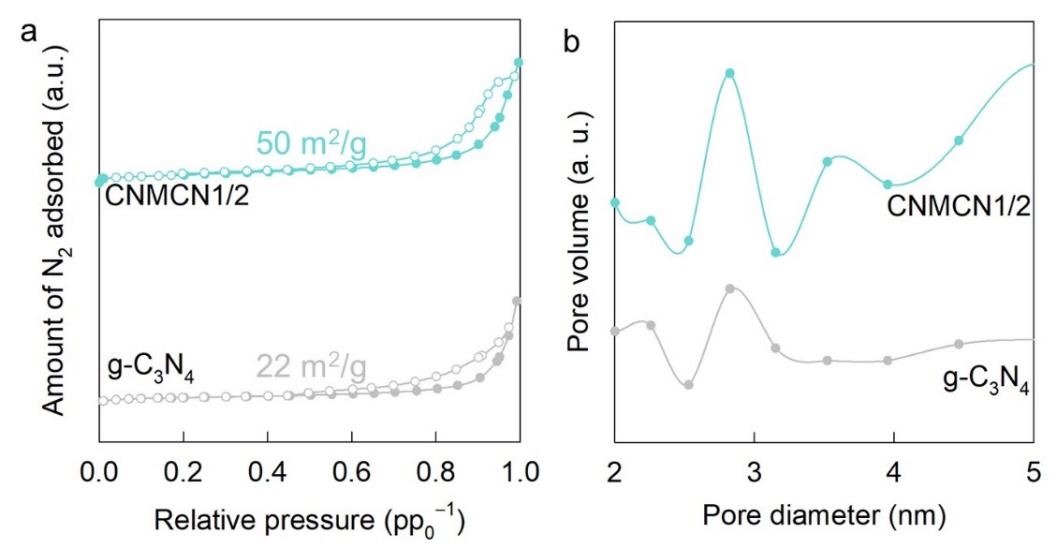
**

**Figure S10**. (a) N_2_ adsorption−desorption isotherm data and (b) pore structure of **CSMCN**1/2 and g-C_3_N_4_.


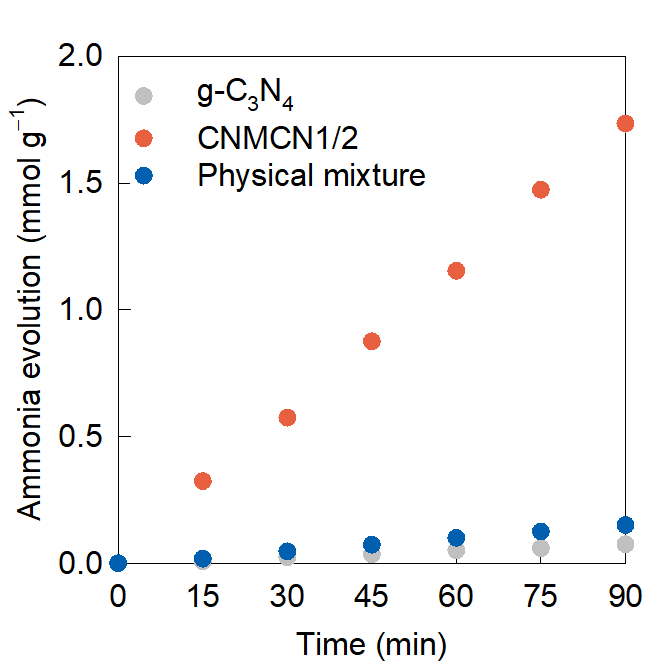


Figure S11. Visible light-induced N_2_ reduction reaction (NRR) of g-C_3_N_4_, CNMCN1/2 and physical mixture of g-C_3_N_4_ and MCN1/2 nanosheets.

Table S10. Recently-reported visible-light (λ > 420 nm)-induced NRR performances of g-C_3_N_4_-based photocatalysts without sacrificial agent.

| Material | Light source | NH_3_ generation rate  (μmol h^−1^ g^−1^) | Ref. |
| --- | --- | --- | --- |
| Alkynyl functionalized g-C_3_N_4_ | 300 W Xe lamp | 120 | [18] |
| PF-g-C_3_N_4_ | 300 W Xe lamp | 82.14 | [19] |
| g-C_3_N_4_-V | 300 W Xe lamp | 54 | [20] |
| Nb_2_O_5_/C/Nb_2_C/g-C_3_N_4_ | 300 W Xe lamp | 365 | [21] |
| SiW_12_/K-C_3_N_4_ | 100mW cm^−2^ Xe lamp | 353.2 | [22] |
| Ru/g-C_3_N_4_ | 300 W Xe lamp | 10.23 | [23] |
| B doped g-C_3_N_4_ | 300 W Xe lamp | 135 | [24] |
| B/CN co-doped g-C_3_N_4_ | 300 W Xe lamp | 116 | [25] |
| COF-g-C_3_N_4_ | 300 W Xe lamp | 236 | [26] |
| Bi_2_WO_6_/g-C_3_N_4_ | 300 W Xe lamp | 452 | [27] |
| B-g-C_3_N_4_ | 300 W Xe lamp | 213 | [28] |
| g-C_3_N_4_−MoS_2_ | 300 W Xe lamp | 582 | [29] |
| PCN-V | 300 W Xe lamp | 1020 | [30] |
| g-C_3_N_4_-N_3c_-0.3 | 300 W Xe lamp | 1086 | [31] |
| g-C_3_N_4_−TiO_2_ | 300 W Xe lamp | 1066 | [32] |
| **CNMCN1/2** | 300 W Xe lamp | 1153 | This work |

**
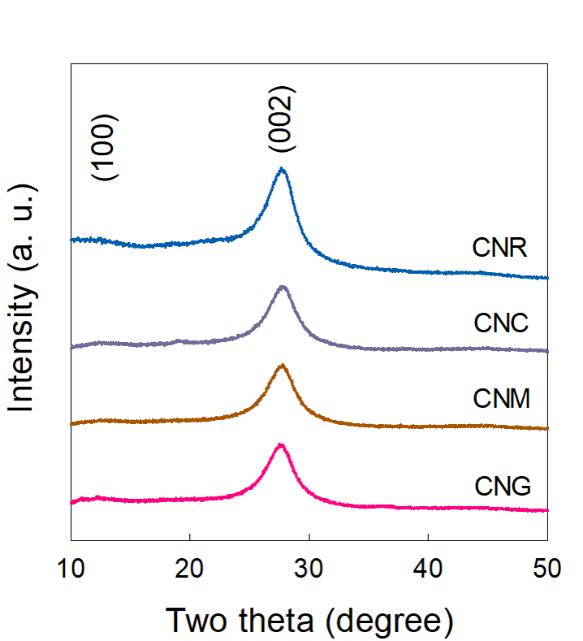
**

Figure S12. Powder XRD patterns of CNR, CNC, CNM, and CNG nanohybrids.


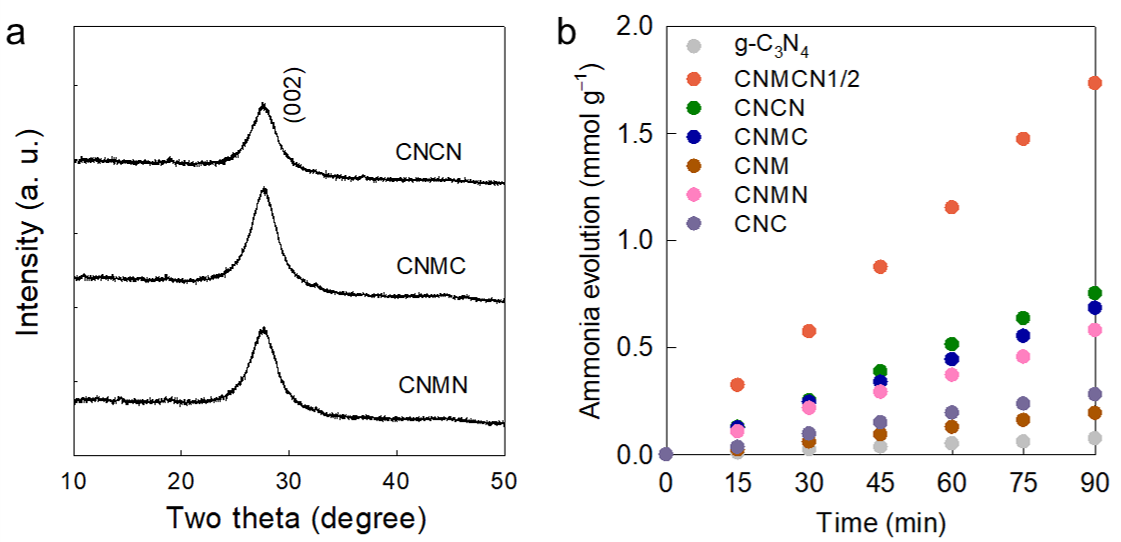


Figure S13. (a) Powder XRD patterns and (b) visible light-induced NRR performance of CNMN, CNMC, and CNCN nanohybrids.

**
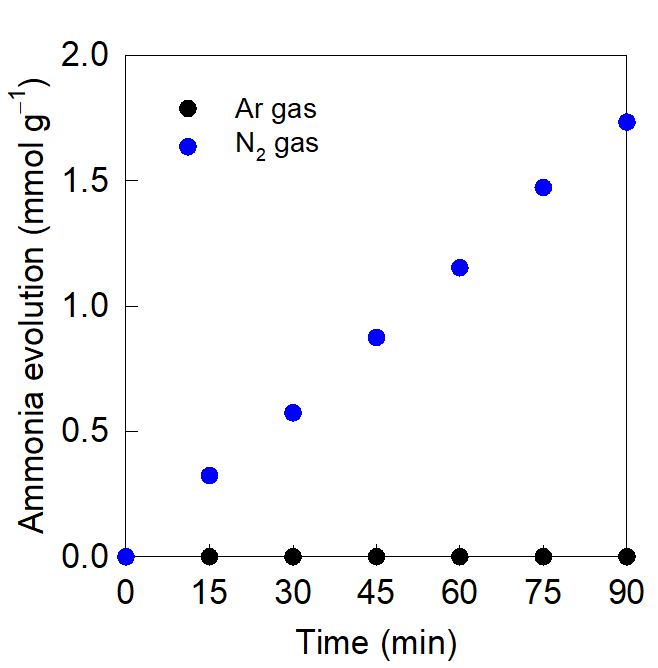
**

Figure S14. Quantitative measurement of NH_4_^+^ generated in different gas atmospheres for CNMCN1/2.


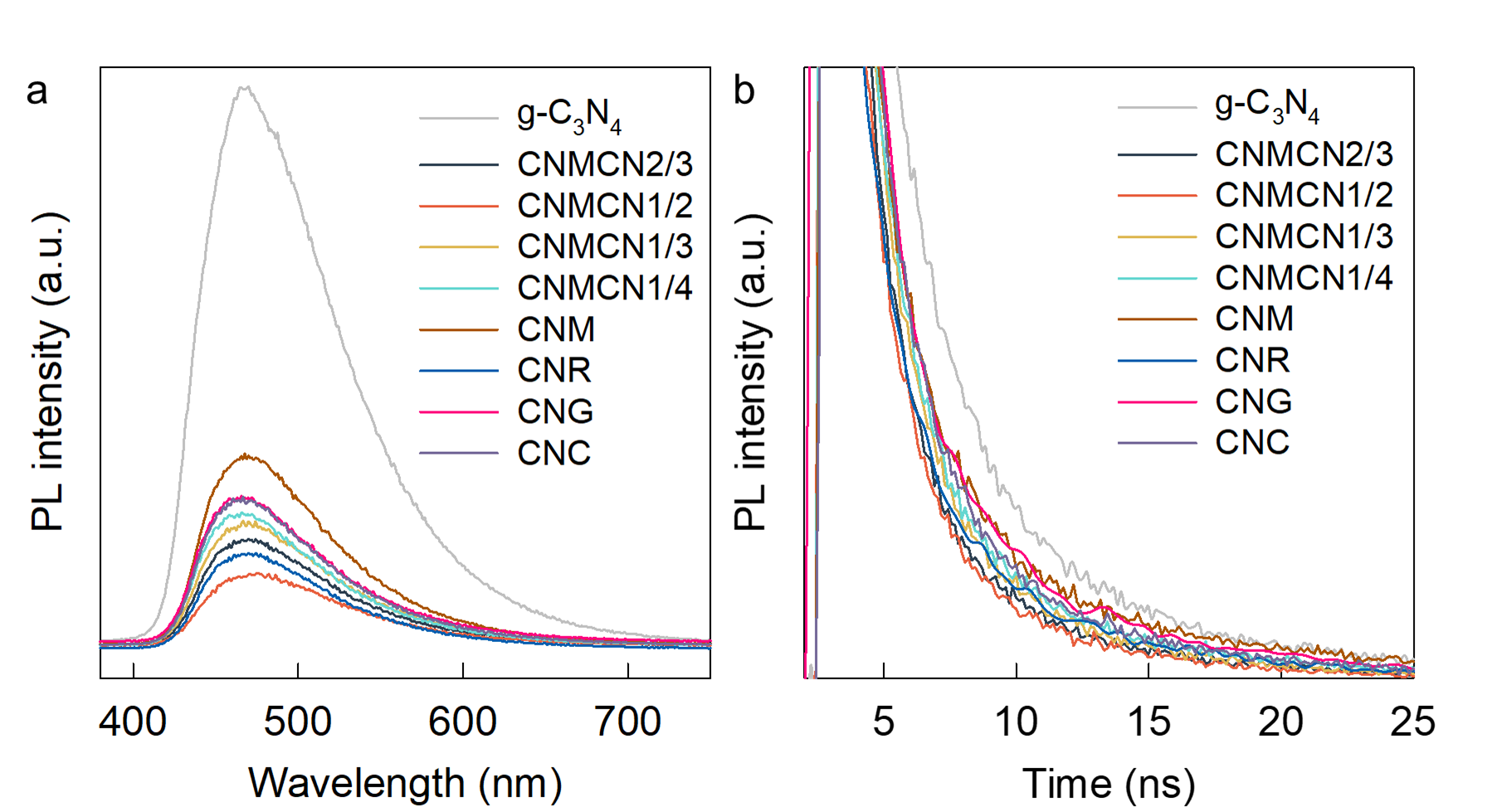


Figure S15. (a) Photoluminescence (PL) spectroscopic spectra and (b) time-resolved PL (TRPL) spectra of CNMCN nanohybrids with several references.


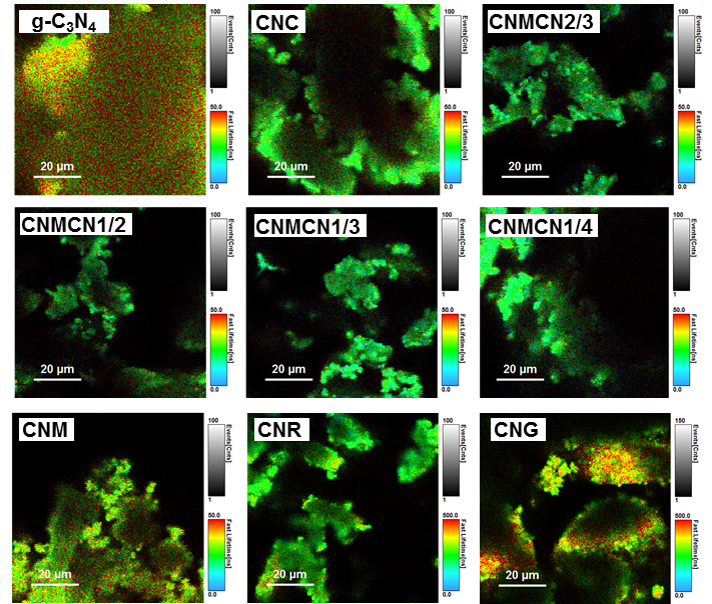


Figure S16. PL lifetime images of CNMCN nanohybrids with several references.

Table S11. PL decay parameters of CNMCN nanohybrids with several references.

| Material | τ_1_ (ns) | A_1_ (%) | τ_2_ (ns) | A_2_ (%) |
| --- | --- | --- | --- | --- |
| g-C_3_N_4_ | 1.78 | 84.3 | 7.6 | 15.7 |
| **CNC** | 1.36 | 88.1 | 7.2 | 11.9 |
| **CNMCN2/3** | 1.27 | 89.2 | 6.6 | 10.8 |
| **CNMCN1/2** | 1.19 | 89.3 | 6.0 | 10.7 |
| **CNMCN1/3** | 1.29 | 88.7 | 6.8 | 11.3 |
| **CNMCN1/4** | 1.30 | 88.4 | 6.9 | 11.6 |
| **CNM** | 1.51 | 87.3 | 7.5 | 12.7 |
| **CNR** | 1.25 | 88.9 | 6.2 | 11.1 |
| **CNG** | 1.45 | 87.6 | 7.4 | 12.4 |


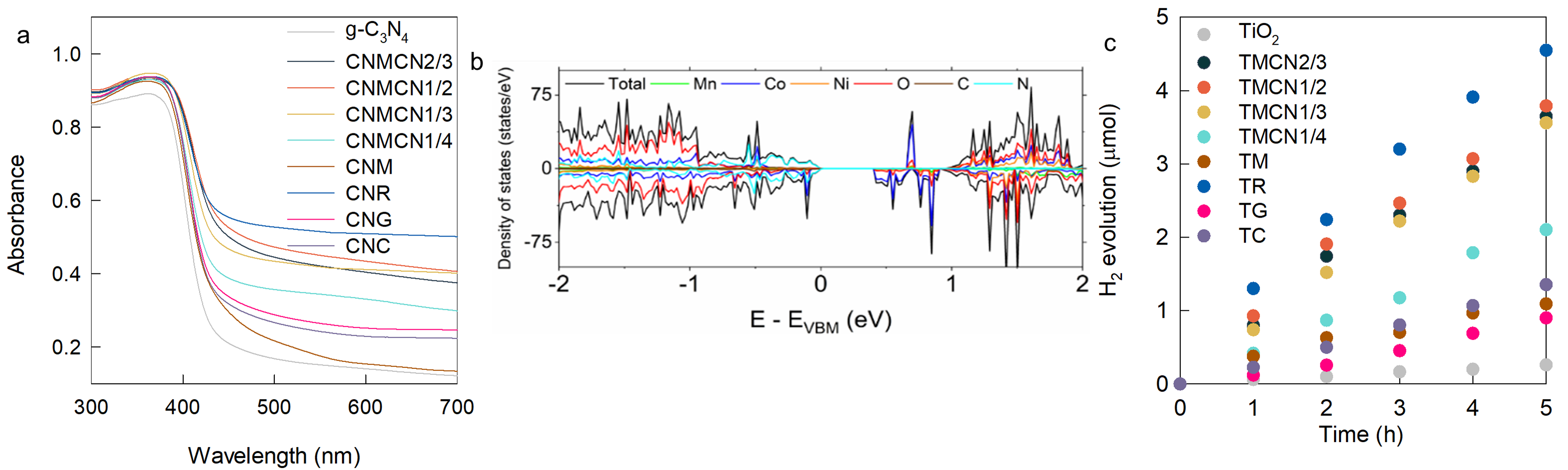


Figure S17. (a) Diffuse reflectance UV−vis spectra of CNMCN nanohybrids with several references, (b) partial density of states (PDOS) of CNMCN1/2, and (c) visible light-induced HER by TiO_2_−[Mn_x_Co_1−2x_Ni_x_]O_2_ nanohybrids with several references.


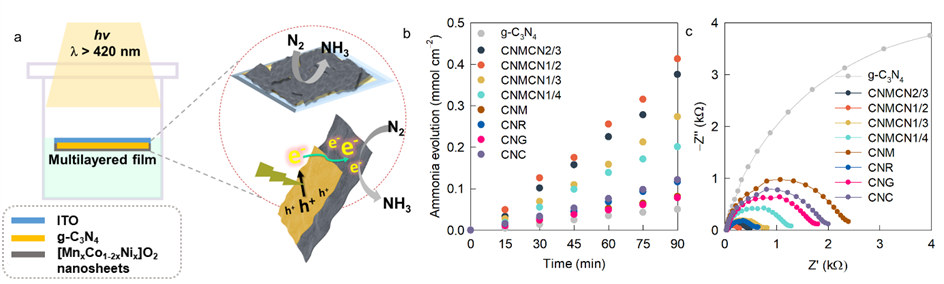


Figure S18. (a) Schematic diagram for the experimental setup for probing the relative efficiency of trimetallic [Mn_x_Co_1−2x_Ni_x_]O_2_ nanosheet as a cocatalyst, (b) cocatalyst efficiency of [Mn_x_Co_1−2x_Ni_x_]O_2_ for visible light-induced NRR, and (c) Nyquist plots of CNMCN nanohybrids with several references.


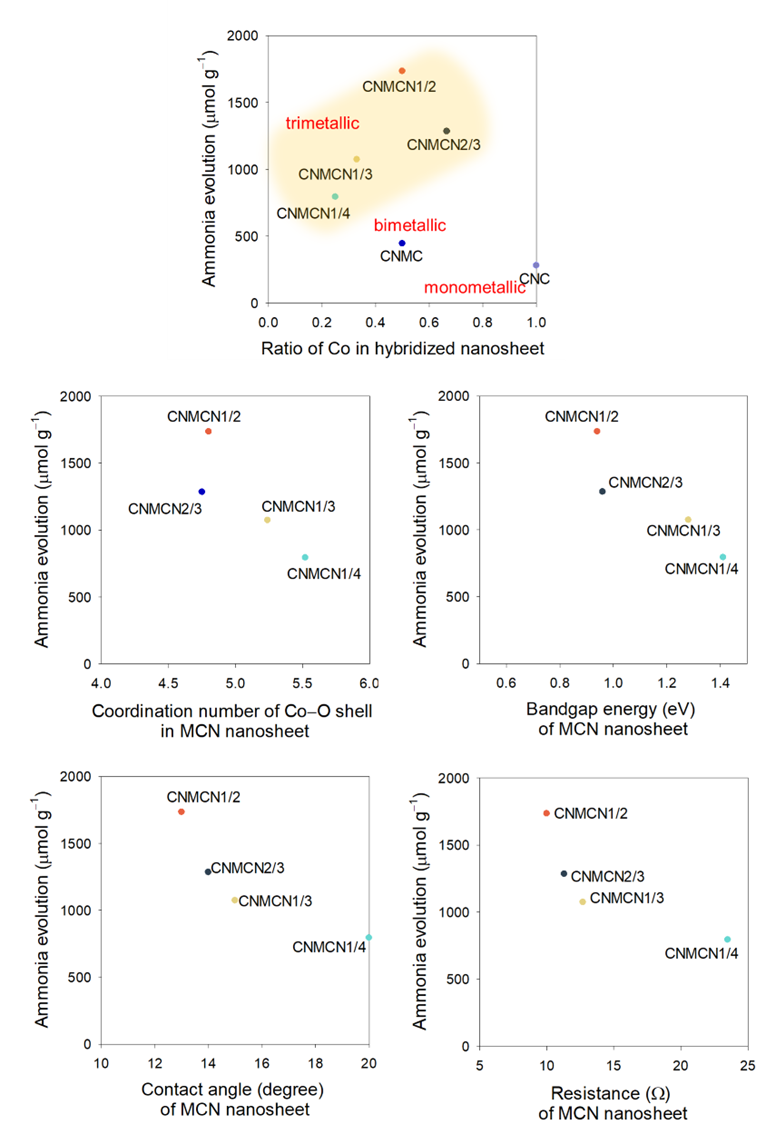


Figure S19. Relationship between several physicochemical features of [Mn_x_Co_1−2x_Ni_x_]O_2_ nanosheets and the photocatalytic performance (ammonia evolution after 1.5 hr) of the CNMCN nanohybrids.


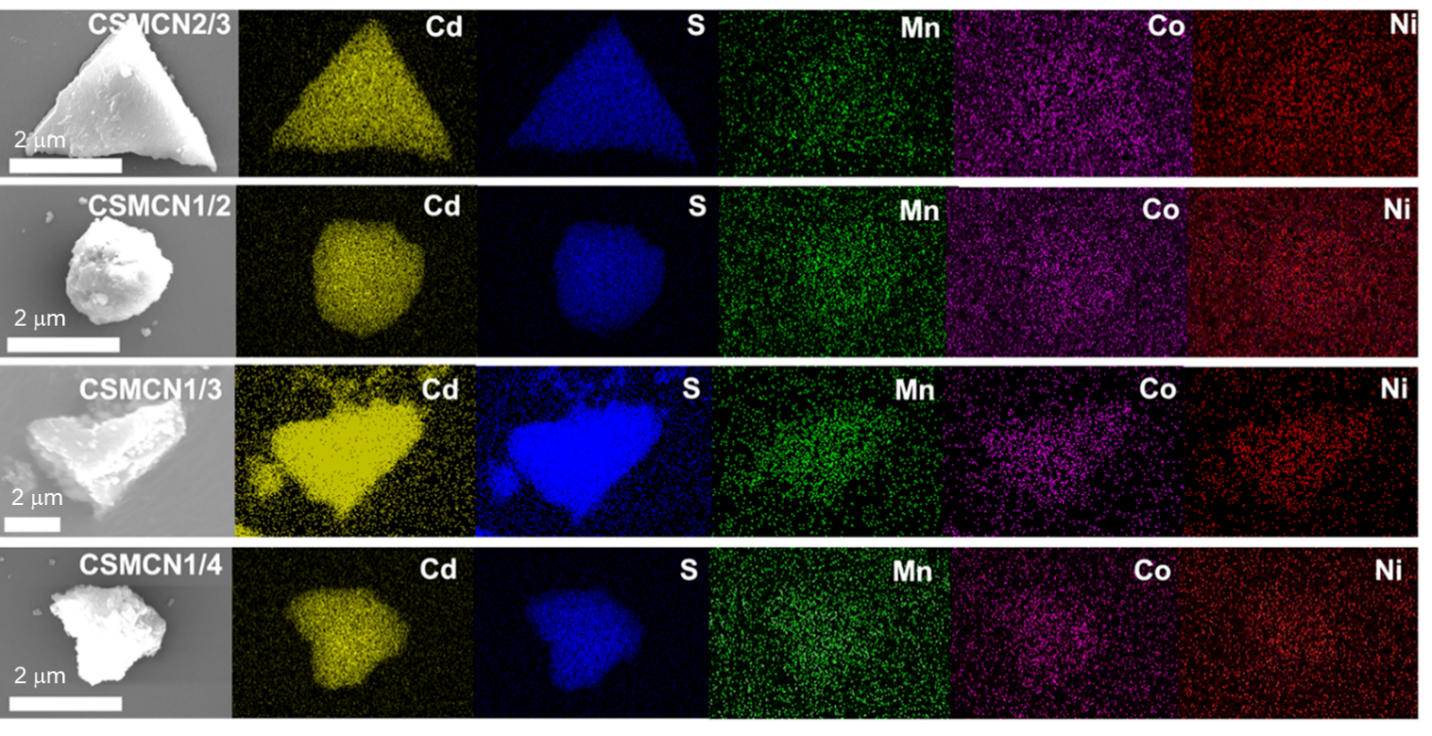


Figure S20. EDS–elemental maps of CSMCN nanohybrids.


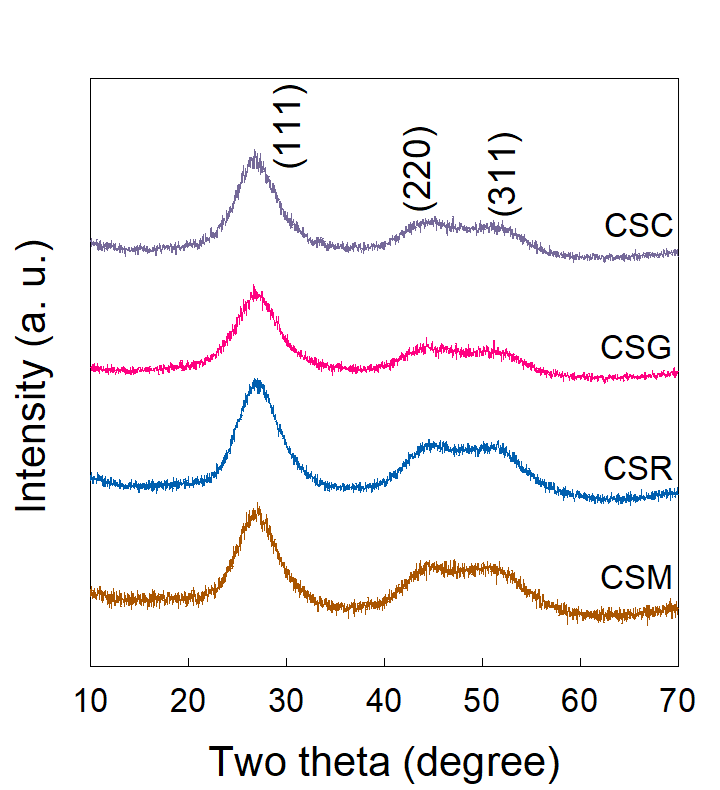


Figure S21. Powder XRD patterns of conductive nanosheet−CdS nanohybrids.


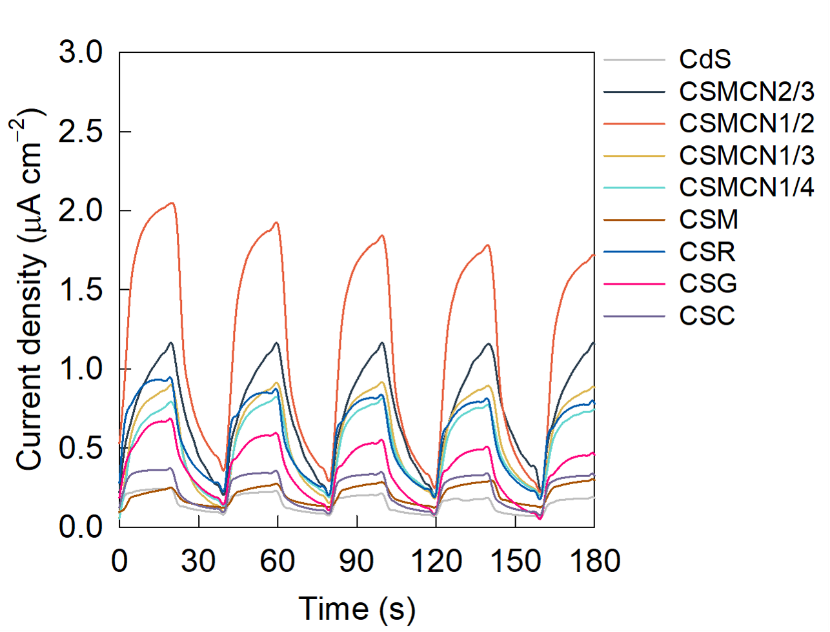


**Figure S22**. Visible light-induced photocurrent generation of CSMCN nanohybrids with several references.


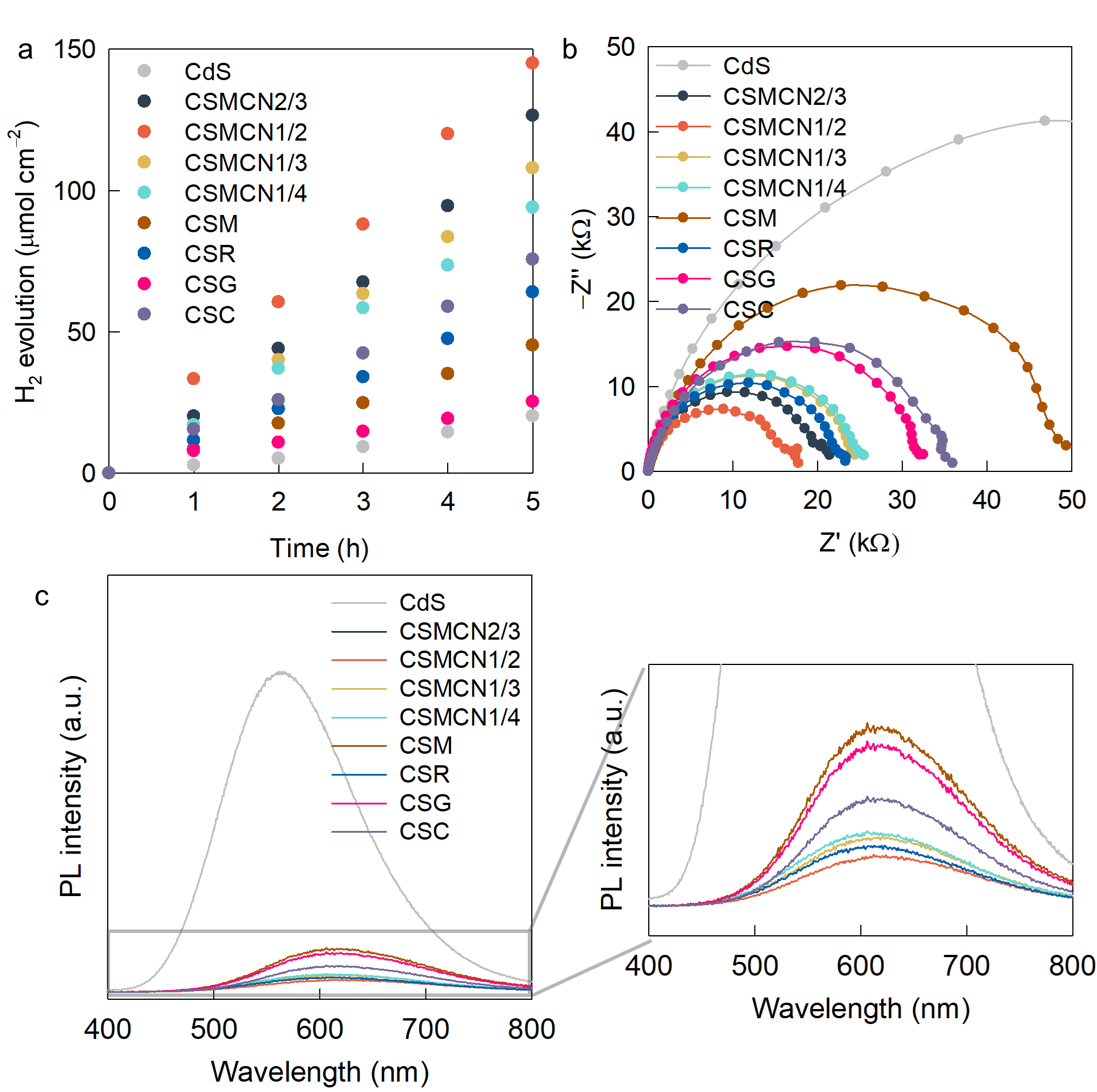


Figure S23. (a) Cocatalyst efficiency of [Mn_x_Co_1−2x_Ni_x_]O_2_ for visible light-induced HER. (b) Nyquist plots and (c) PL spectroscopic analysis of CSMCN nanohybrids with several references.


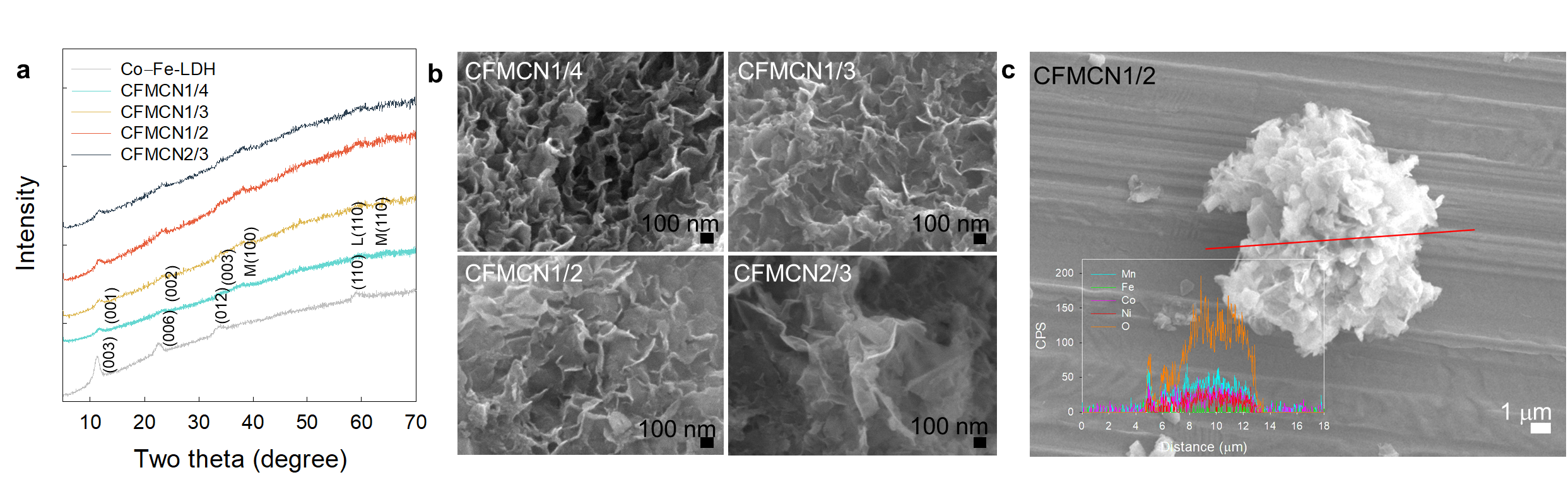


Figure S24. (a) XRD and (b) FE-SEM images of CFMCN nanohybrids. (c) EDS−line mapping data of CFMCN1/2.


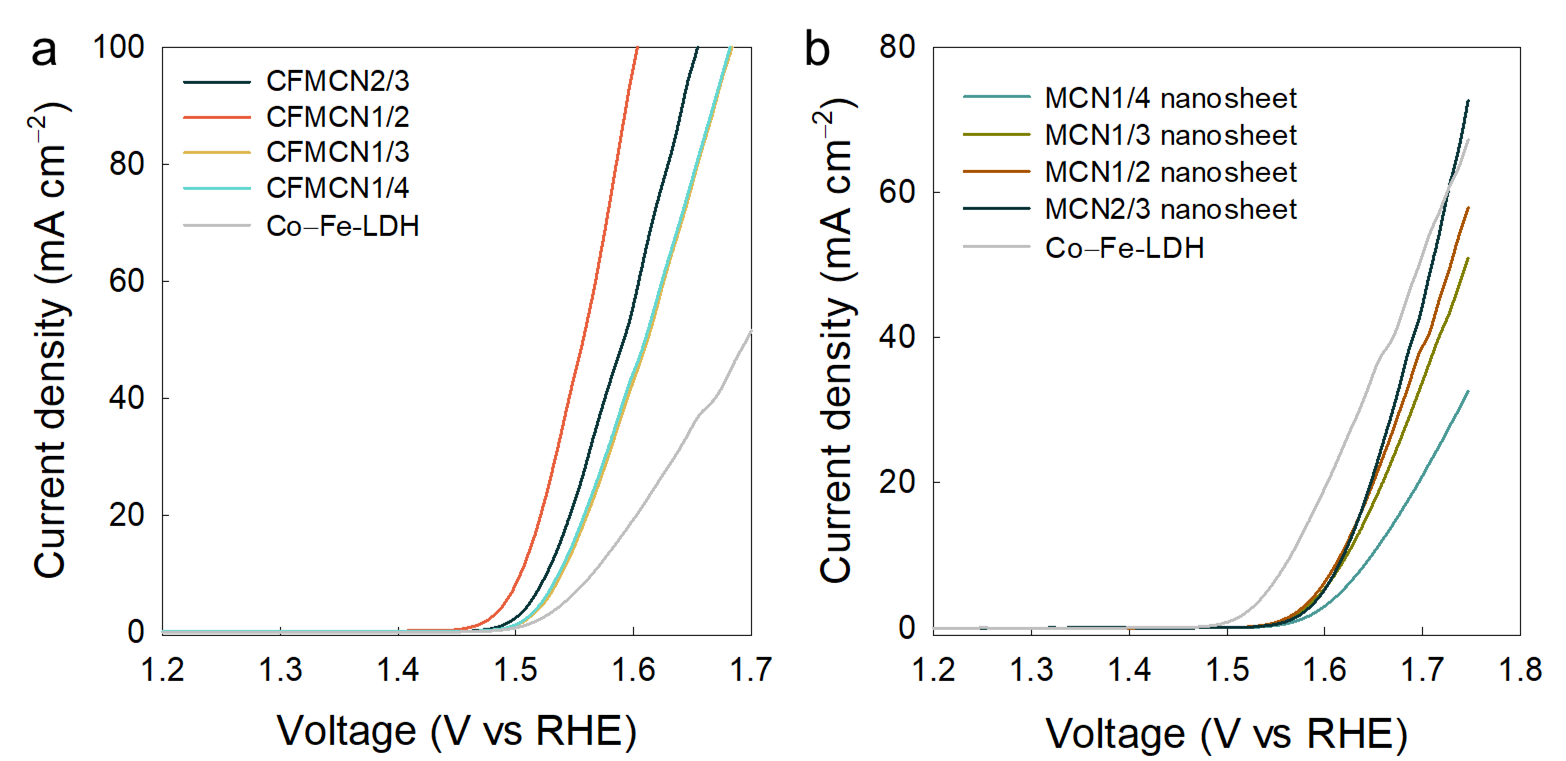


Figure S25. Linear sweep voltammetry (LSV) curves of (a) Co−Fe-LDH and CFMCN nanohybrids, and (b) MCN nanosheets.


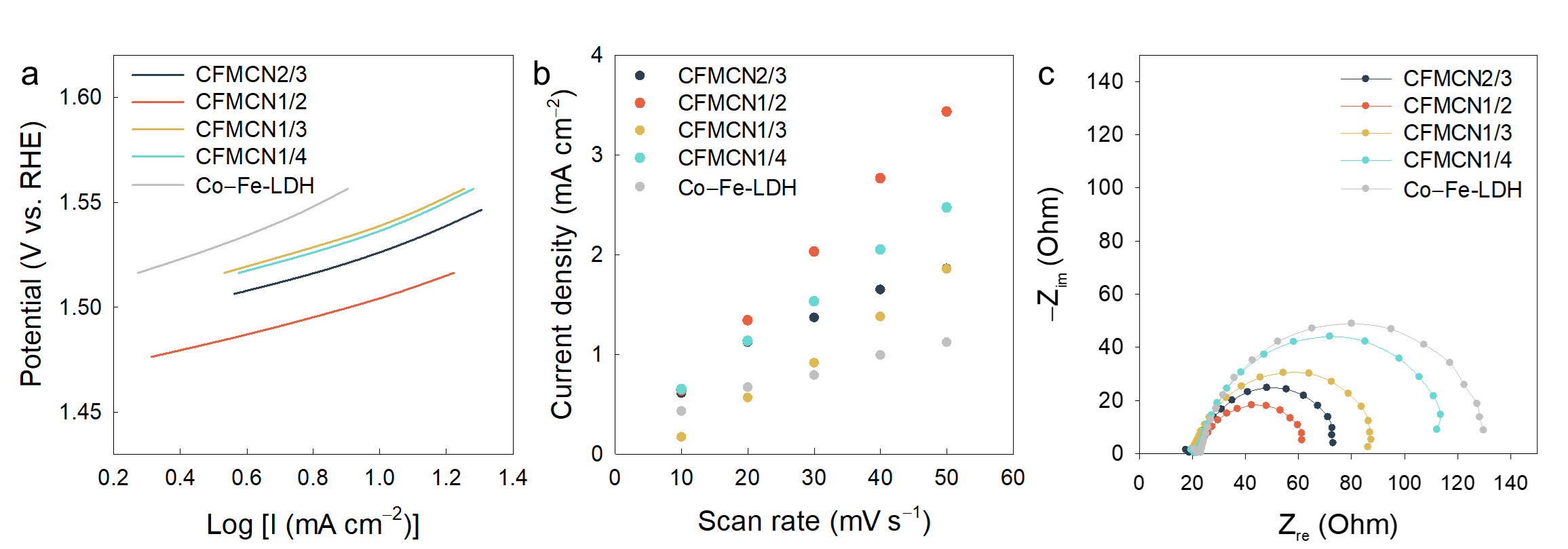


Figure S26. (a) Tafel slopes, (b) plots of charging current density differences versus scan rates, and (c) Nyquist plots of Co−Fe-LDH and CFMCN hybrids.

Table S12. Oxygen evolution reaction (OER) electrocatalyst performances of Co−Fe-LDH and CFMCN nanohybrids in 1.0 M KOH electrolyte.

| Sample | Overpotential  @ 10 mA cm^−2^ (mV) | Tafel value  (mV dec^−1^) | Double layer capacitance (C_dl_)  (mF cm^−2^) | Charge transfer resistance (Ω) |
| --- | --- | --- | --- | --- |
| Co−Fe-LDH | 335 | 63.0 | 17.0 | 119 |
| **CFMCN1/4** | 305 | 56.1 | 56.8 | 92 |
| **CFMCN1/3** | 309 | 54.8 | 41.8 | 65 |
| **CFMCN1/2** | 271 | 43.7 | 78.0 | 40 |
| **CFMCN2/3** | 296 | 53.4 | 43.2 | 53 |

^
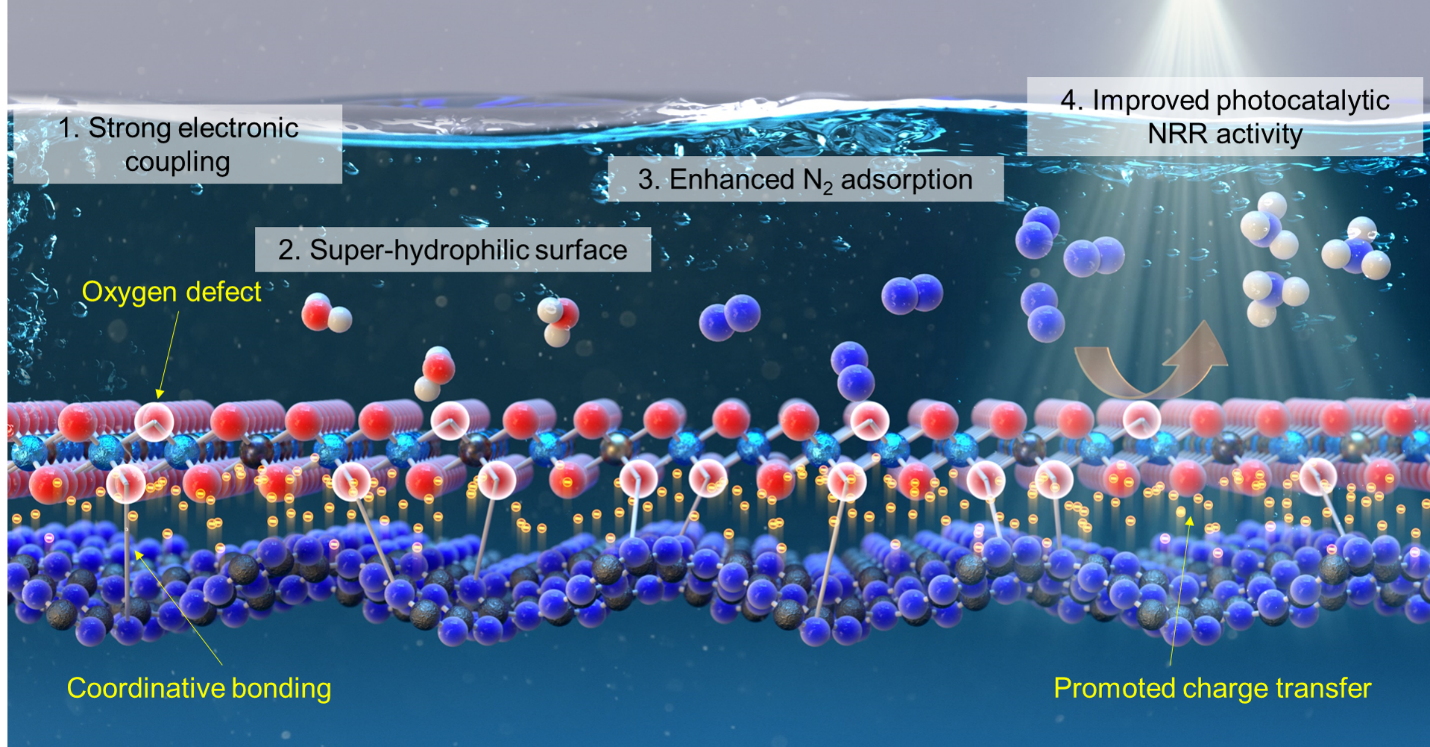
^

Figure S27. Electronic coupling effects of CNMCN1/2 nanohybrid.

Table S13. K-point mesh for DFT calculations.

| System | k-mesh | Scheme |
| --- | --- | --- |
| CoO_2_ | 9×9×1 | Γ-centered |
| **MCN2/3** | 4×5×1 | Γ-centered |
| **MCN1/2** | 5×5×1 | Γ-centered |
| **MCN1/3** | 3×9×1 | Γ-centered |
| **MCN1/4** | 2×5×1 | Γ-centered |
| MnO_2_ | 9×9×1 | Γ-centered |
| g-C_3_N_4_ | 6×6×1 | Γ-centered |

**References**

[1] E.-J. Oh, T. W. Kim, K. M. Lee, M.-S. Song, A.-Y. Jee, S. T. Lim, H.-W. Ha, M. Lee, J.-H. Choy, S.-J. Hwang, *ACS Nano* **2010**, *4*, 4437.

[2] N. H. Kwon, J. Park, X. Jin, S.-J. Kim, H. Kim, S.-J. Hwang, *ACS Nano* **2023**, *17*, 23732.

[3] N. H. Kwon, J. M. Lee, T.-H. Gu, X. Jin, S.-J. Hwang, *Solar RRL* **2021**, *5*, 2000411.

[4] X. Jin, D. A. Agyeman, S. Kim, Y. H. Kim, M. G. Kim, Y.-M. Kang, S.-J. Hwang, *Nano Energy* **2020**, *67*, 104192.

[5] S. Son, J. M. Lee, S.-J. Kim, H. Kim, X. Jin, K. K. Wang, M. Kim, J. W. Hwang, W. Choi, Y.-R. Kim, H. Kim, S.-J. Hwang, *Appl. Catal. B: Environ.* **2019**, *257*, 117875.

[6] G. Kresse, J. Furthmüller, *J. Phys. Rev. B* **1996**, *54*, 11169.

[7] P. E. Blöchl, *Phys. Rev. B* **1994**, *50*, 17953.

[8] J. P. Perdew, K. Burke, M. Ernzerhof, *Phys. Rev. Lett.* **1996**, *77*, 3865.

[9] S. Grimme, J. Antony, S. Ehrlich, H. Krieg, *J. Chem. Phys*. **2010**, *132*, 154104.

[10] H. J. Monkhorst, J. D. Pack, *Phys. Rev. B* **1976**, *13*, 5188.

[11] B. Farkaš, D. Santos-Carballal, A. Cadi-Essadek, N. H. de Leeuw, *Materialia* **2019**, *7*, 100381.

[12] L. M. Morgan, M. M. Islam, H. Yang, K. O’Regan, A. N. Patel, A. Ghosh, E. Kendrick, M. Marinescu, G. J. Offer, B. J. Morgan, M. S. Islam, J. Edge, A. Walsh, *ACS Energy Lett.* **2022**, *7*, 108.

[13] M. Yu, D. R. Trinkle, *J. Chem. Phys.* **2011**, *134*, 064111.

[14] Y. Koyama, H. Arai, I. Tanaka, Y. Uchimoto, Z. Ogumi, *J. Mater. Chem. A* **2014**, *2*, 11235.

[15] K. Mathew, R. Sundararaman, K. Letchworth-Weaver, T. A. Arias, R. G. Henning, *J. Chem. Phys.* **2014**, *140*, 084106.

[16] J. K. Nørskov, J. Rossmeisl, A. Logadottir, L. Lindqvist, J. R. Kitchin, T. Bligaard, H. Jonsson, *J. Phys. Chem. B* **2004**, *108*, 17886.

[17] W. M. Hayness, CRC handbook of chemistry and physics, CRC press, Florida, USA 2014.

[18] W. Li, M. Sun, Z. Ding, Q. Zeng, Y. Zheng, W. Sun, X. Meng, *Sep. Purif. Technol.* **2022**, *282*, 120097.

[19] Y. Li, M. Ti, D. Zhao, Y. Zhang, L. Wu, Y. He, *J. Alloys Compd.* **2021**, *25*, 159298.

[20] Y. Zhang, J. di, P. Ding, J. Zhao, K. Gu, X. Chen, C. Yan, S. Yin, J. Xia, H. Li, *J. Colloid Interface Sci.* **2019**, *553*, 530.

[21] H. Jiang, C. Zhang, Y. Zhang, W. Wang, C. Yang, B. Sun, Y. Shen, F. Bian, *Catal. Sci. Technol.* **2020**, *10*, 5964.

[22] C. Xiao, L. Zhang, K. Wang, H. Wang, Y. Zhou, W. Wang, *Appl. Catal. B: Environ.* **2018**, *239*, 260.

[23] S. Wang, D. Guo, M. Zong, C. Fan, J. Xu, D.-H. Wang, *Appl. Catal. A: Gen.* **2021**, *617*, 118112.

[24] Y. Chen, M. Yu, G. Huang, Q. Chen, J. Bi, *Small* **2022**, *18*, 2205388.

[25] D. Sun, L. Li, Y. Yu, L. Huang, F. Meng, Q. Su, S. Ma, B. Xu, *J. Colloid Interfaces Sci.* **2021**, *600*, 639.

[26] T. Shan, H. Luo, S. Wu, J. Li, F. Zhang, H. Xiao, L. Huang, L. Chen, *Fuel* **2024**, *358*, 130157.

[27] E. Dhanaraman, A. Verma, P.-H. Chen, N.-D. Chen, Y. Siddiqui, Y.-P. Fu, *Sol. RRL* **2024**, *8*, 2300981.

[28] J. Lee, S. H. W. Kok, B.-J. Ng, X. Y. Kong, L. K. Putri, S.-P. Chai, L.-L. Tan, *J. Environ. Chem. Eng.* **2023**, *11*, 109511.

[29] N. H. Kwon, S.-J. Shin, X. Jin, Y. Jung, G.-S. Hwang, H. Kim, S.-J. Hwang, *Appl. Catal. B: Environ.* **2021**, *277*, 119191.

[30] Y. Shiraishi, S. Shiota, Y. Kofuji, M. Hashimoto, K. Chishiro, H. Hirakawa, S. Tanaka, S. Ichikawa, T. Hirai, *ACS Appl. Energy Mater.* **2018**, *1*, 4169.

[31] Y. Xue, C. Ma, Q. Yang, X. Wang, S. An, X. Zhang, J. Tian, *Chem. Eng. J.* **2023**, *457*, 141146.

[32] N. H. Kwon, J. Park, X. Jin, S.-J. Kim, H. Kim, S.-J. Hwang, *ACS Nano* **2023**, *17*, 23732.
